# Atomic structure of the human herpesvirus 6B capsid and capsid-associated tegument complexes

**DOI:** 10.1038/s41467-019-13064-x

**Published:** 2019-11-25

**Authors:** Yibo Zhang, Wei Liu, Zihang Li, Vinay Kumar, Ana L. Alvarez-Cabrera, Emily C. Leibovitch, Yanxiang Cui, Ye Mei, Guo-Qiang Bi, Steve Jacobson, Z. Hong Zhou

**Affiliations:** 1 Center for Integrative Imaging, Hefei National Laboratory for Physical Sciences at the Microscale, and School of Life Sciences, University of Science and Technology of China (USTC), Hefei, Anhui 230026 China; 2 California NanoSystems Institute, University of California, Los Angeles (UCLA), Los Angeles, CA 90095-7151 USA; 3 Department of Microbiology, Immunology and Molecular Genetics, UCLA, Los Angeles, CA 90095-7364 USA; 4 State Key Laboratory of Precision Spectroscopy, School of Physics and Electronic Science, East China Normal University (ECNU), Shanghai, 200062 China; 5 Viral Immunology Section, National Institute of Neurological Disorders and Stroke, National Institutes of Health (NIH), Bethesda, MD 20892 USA

**Keywords:** Cryoelectron microscopy, Biological techniques

## Abstract

Human herpesvirus 6B (HHV-6B) belongs to the β-herpesvirus subfamily of the *Herpesviridae*. To understand capsid assembly and capsid-tegument interactions, here we report atomic structures of HHV-6B capsid and capsid-associated tegument complex (CATC) obtained by cryoEM and sub-particle reconstruction. Compared to other β-herpesviruses, HHV-6B exhibits high similarity in capsid structure but organizational differences in its CATC (pU11 tetramer). 180 “VΛ”-shaped CATCs are observed in HHV-6B, distinguishing from the 255 “Λ”-shaped dimeric CATCs observed in murine cytomegalovirus and the 310 “Δ”-shaped CATCs in human cytomegalovirus. This trend in CATC quantity correlates with the increasing genomes sizes of these β-herpesviruses. Incompatible distances revealed by the atomic structures rationalize the lack of CATC’s binding to triplexes Ta, Tc, and Tf in HHV-6B. Our results offer insights into HHV-6B capsid assembly and the roles of its tegument proteins, including not only the β-herpesvirus-specific pU11 and pU14, but also those conserved across all subfamilies of *Herpesviridae*.

## Abstract (web summary)

Human Herpesvirus 6B (HHV-6) can cause fever, diarrhea and roseola rash. Here the authors present a cryoEM approach to image crude, minimally purified virus samples and employ it to determine the atomic structures of HHV-6B capsid and capsid-associated tegument protein complexes.

## Introduction

First discovered in 1986 as a member of the β-herpesvirus subfamily of *Herpesviridae*^1^, human herpesvirus 6 (HHV-6) is now understood to be a set of two closely related herpesvirus species known as HHV-6A and HHV-6B. HHV-6 infects nearly all human beings by the age of three and often results in fever, diarrhea, and the roseola rash. Herpesviruses—such as herpes simplex, chicken pox, and the Epstein–Barr virus—have a tendency of establishing life-long latency, activating later in life with many clinical manifestations; HHV-6 follows a similar cycle. HHV-6 reactivation in brain tissue can cause cognitive dysfunction, permanent disability, and death. Recent studies have suggested a link between HHV-6 and formation of the Alzheimer’s disease-associated β-amyloid and a subset of refractory epilepsy^2,3^.

Difficulties in cultivating HHV-6B have prevented high-resolution structural studies and limited our understanding of its assembly. Despite recent progress in our understanding of representative members of all three subfamilies of the *Herpesviridae*^4–8^, including fellow β-herpesvirus subfamily members such as human^8^ and murine^9^ cytomegaloviruses, the only available structures for HHV-6 are a very low-resolution (30 Å) capsid structure, generated from ~ 30 particles^10^, and a crystal structure for pU14 (a putative tegument protein with unknown functions)^11^. However, human cytomegalovirus (HCMV) has its structure established at a resolution of 3.9 Å; it shows that pp150, a β-herpesvirus subfamily-specific tegument protein, forms a “△”-shaped group-of-three structure on each of the 320 triplexes in a capsid, leading to a net that encloses the capsid. Such capsid-associated tegument complexes (CATCs) may act, in addition to sensing host states^12^ and other possible functions, to secure the underlying HCMV capsid after packaging its genome of 235 kb^8^, which is the largest among all human herpesviruses (in comparison, the smallest genome size is 120 kb for the genome of α-herpesvirus Varicella-zoster virus). Given that HHV-6B has a genome size of 162 kb—much smaller than that of HCMV—the question arises whether its β-herpesvirus-specific tegument protein, pU11, binds capsids in the same way as HCMV’s homologous protein pp150/pUL32 does. If so, it would challenge the previously suggested role of pp150/pUL32 maintaining capsid stability after being pressurized by HCMV’s large genome^8^.

Here, we have employed a plethora of cutting-edge cryoEM techniques and a sub-particle reconstruction method to work with very little and minimally purified HHV-6B sample and obtained the near-atomic resolution structure of HHV-6B. We have derived atomic models for a total of 59 conformers of the four capsid proteins and one tegument protein of HHV-6B. Our results show a capsid-binding pattern of pU11 that differs from CATC binding patterns found in HCMV and murine cytomegalovirus (MCMV). Our observation of pU11’s absence from atop triplexes Ta, Tc, and Tf and its unique capsid-binding pattern offers major insights into the roles of not only the β-herpesvirus-specific pU11 (pUL32/pp150) CATCs but also CATCs across all subfamilies of *Herpesviridae*.

## Results

### Organization of the dsDNA genome inside HHV-6B

HHV-6B is known to be cell-associated and grows at very low titer compared with other β-herpesviruses such as cytomegaloviruses, presenting a major challenge in isolating high-concentration samples for structural studies over the last 15 years since first attempted^10^. We circumvented these limitations by employing a combination of advanced imaging technologies^13–15^ to precisely target sparsely distributed HHV-6B virions (~ 1.5 particles per movie, Supplementary Fig. 1a, b), and acquired a total of 6443 high-quality virion particle images for cryoEM icosahedral reconstruction.

**Fig. 1:**
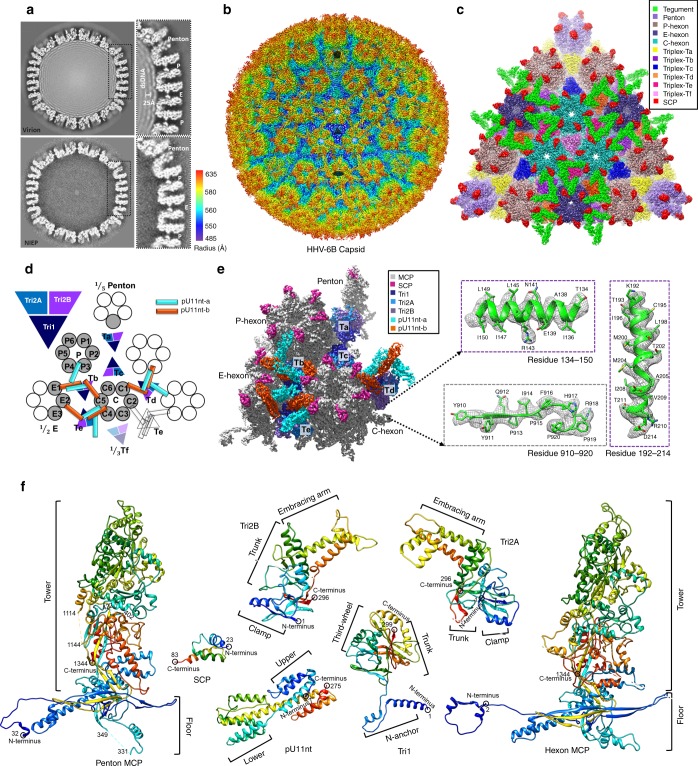
The 3D reconstruction and atomic models of HHV-6B capsid and CATC. **a** Density slices of HHV-6B virion (top) and noninfectious enveloped particle (NIEP, bottom) reconstructions at ~ 9 Å resolutions, with zoomed-in boxed subsection showing a ~ 25 Å inter–DNA duplex distance. **b** Radially colored cryoEM density map of the HHV-6B icosahedral reconstruction, viewed along a threefold axis. Only the icosahedral symmetric components, including the capsid and capsid-associated tegument complex (CATC), are visible. The shaded pentagon, triangle, and oval mark the fivefold, threefold, and twofold axis, respectively. **c** Zoomed-in surface view of one facet of the icosahedral capsid with structural components colored as indicated in the legend. Density of triplex Tf region at the center is smeared owing to imposition of threefold symmetry during icosahedral reconstruction. A slightly lower density threshold was used for the pentons and triplexes Tf such that their volumes are comparable to subunits elsewhere. Triplexes Ta, Tc, and Tf all lack tegument protein associations. **d** Schematic of one asymmetric unit (gray shade) of the reconstruction of the capsid, with individual capsid protein subunits labeled following the HCMV nomenclature^8^. Subunits of triplex Tf are shown in semi-transparent colors instead of solid ones because their orientations were not determined in the icosahedral reconstruction. **e** CryoEM density map of one asymmetric unit of the reconstruction, with individual capsid protein subunits labeled. The right panels show close-up views of the cryoEM density map (gray mesh) of a loop (box labeled “Residues 910–920”) in the MCP tower region and two helices (boxes labeled “Residues 134–150” and “Residues 192–214”) in the Tri2B trunk and embracing arm domains, respectively, superposed with their atomic models, illustrating side-chain features. **f** Atomic models of individual capsid (penton and hexon MCP/SCP, Tri1, Tri2A, and Tri2B) and N-terminal 1/3 portion of capsid-associated tegument (pU11nt) proteins, shown as rainbow-gradient ribbons (blue at the N terminus to red at the C-terminus). The models are displayed such that the top corresponds to the outside the virus and the bottom inside the virus. Individual domains in the triplex subunits are labeled.

We obtained an icosahedral reconstruction of 9 Å resolution from 423 DNA-devoid noninfectious enveloped particles (NIEP) and compared it with our HHV-6B virion icosahedral reconstruction (5.1 Å), filtered to 9 Å resolution (Fig. 1a, b). This comparison reveals that the HHV-6B NIEP and virion have nearly identical capsid structures, but differ in their interior compositions [dsDNA is found only in the virion (Fig. 1a)].

In the HHV-6B virion, neighboring dsDNA duplexes are spaced ~ 25 Å apart (Fig. 1a, upper panel). This distance is larger than that of HCMV (~ 23 Å)^8,16^ but comparable with those of HSV-1 (~ 25 Å)^17^ and KSHV (~ 26 Å)^18,19^, consistent with the need to pack their respective genomes of different sizes into capsids of similar space. Most notably, the dsDNA inside HHV-6B is relatively smooth (Fig. 1a, upper panel), whereas the dsDNA in HCMV appears to have been “squeezed,” even appearing in the hexon channels;^8^ this difference is likely also a result of their respective genome sizes.

### Pushing resolution by sub-particle reconstruction

The *T* = 16 icosahedral HHV-6B capsid structure (Supplementary Movie 1) contains 12 pentons, 150 hexons, and 320 triangular triplexes. Notably, the 3D reconstruction shows an unexpected pattern of 180 tegument protein pU11 tetramers bound to the virion capsid (Fig. 1c, d). This reconstruction reveals the molecular boundaries among these proteins and allows us to identify individual molecules (Fig. 1e). (Note: one of the 12 icosahedral vertices of the herpesvirus capsid should be occupied by a DNA packaging/ejection portal complex (referred to as “portal vertex” below) instead of a penton but is not resolved here due to imposition of icosahedral symmetry^20–24^. Nonetheless, based on observations from HSV-1, 12 penton-containing, portal-less capsids are also known to exist and can be obtained by co-expressing the capsid proteins in the absence of the portal protein^25,26^.) Each of the 60 asymmetric units of the HHV-6B capsid contains 16 copies of the major capsid protein (MCP); 16 copies of the smallest capsid protein (SCP), each atop an MCP; five and one-third triplexes (Ta, Tb, Tc, Td, Te, and one-third of Tf); and 12 copies of pU11. Only the N-terminal one-third of pU11, referred to as pU11nt below, is resolved in the cryoEM density map, indicating the rest of the protein is flexible. The 12 pU11nt monomers in each asymmetric unit cluster into three groups-of-four, or tetramers. A pU11nt tetramer binds atop triplex Tb, Td, or Te, but not Ta, Tc, and Tf (Fig. 1d, e).

To obtain higher resolution HHV-6B structures sufficient for atomic modeling, we further processed these virion images through an icosahedral reconstruction-guided sub-particle reconstruction method with local defocus calibration^5,27–30^ to alleviate the depth-of-focus/Ewald sphere curvature limitation^8,31^ and improved the structures of the sub-particles encompassing the icosahedral fivefold, threefold, and twofold axis to resolutions of −3.82 Å, 3.77 Å, and 3.77 Å, respectively (Supplementary Fig. 1c–g and Supplementary Movies 2–4). Local resolution assessments indicate that the capsid shell region in these three sub-particle maps have resolutions uniformly better than 4 Å, whereas the dsDNA densities have much poorer resolutions (Supplementary Figs. 2–4). These density maps have clear features of amino-acid side chains, enabling atomic modeling (Supplementary Figs. 5–8). Remarkably, the number of particles used here is much smaller than those used in previous near-atomic resolution structures of human herpesvirus particles^4–8^, yet we were still able to obtain high-resolution reconstructions (Supplementary Table 1). This indicates the effectiveness of our novel approach of using minimally purified virions and sub-particle reconstruction with local focus recalculation.

From these high-resolution sub-particle reconstructions, we built atomic models for a total of 59 unique conformers of the four capsid proteins and one capsid-associated tegument protein (see examples in Fig. 1f), including 15 hexon MCPs, 1 penton MCP, 15 hexon SCPs, 1 penton SCP, 5 copies of the triplex monomer protein (Tri1), 10 copies of the triplex dimer protein (Tri2), and 12 copies of the pU11nt. In total, all 59 proteins amounted to over 29,000 amino-acid residues.

### Atomic structures of penton and hexon capsomers

**Fig. 2:**
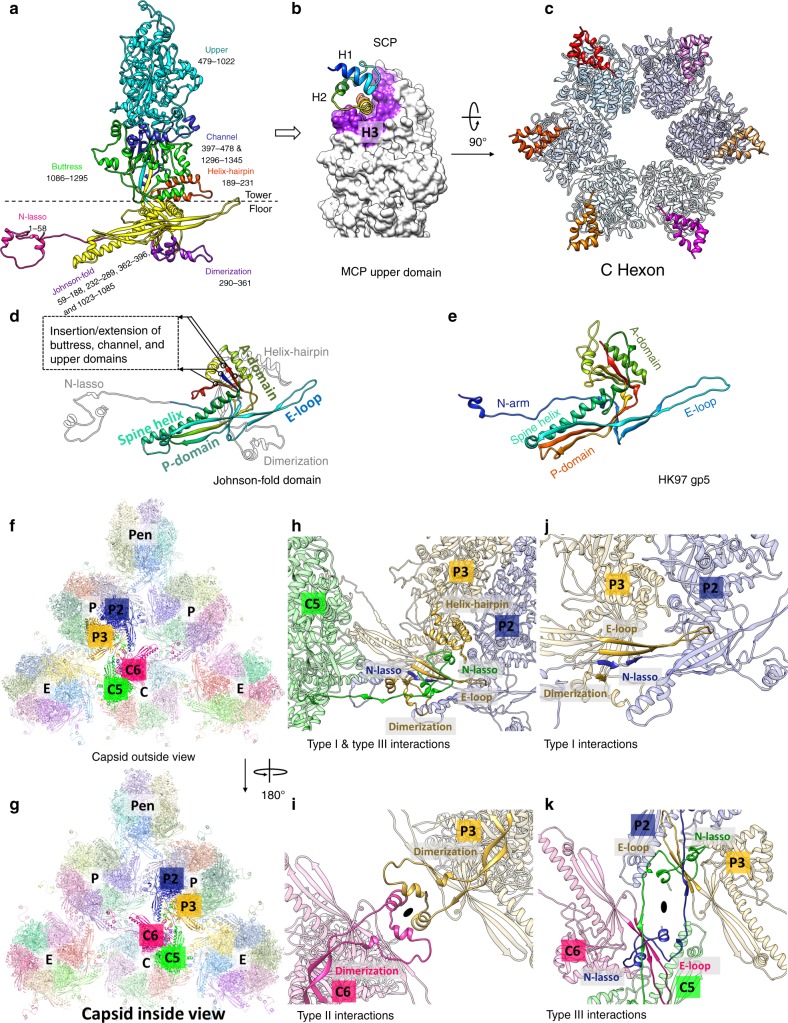
Structures of MCP and SCP, and MCP–MCP and MCP-SCP interactions. **a** Ribbon model of MCP from hexon subunit C6, showing its domain organization. **b**, **c** SCP (ribbons) bound to the MCP upper domain (surface), with SCP-binding groove of MCP highlighted in purple **b**. Six SCPs sit atop a hexon capsomer, as seen from the top of a C hexon **c**. **d**, **e** Structure of the Johnson-fold domain in the HHV-6B MCP floor region (**d**), with helix-hairpin, N-lasso, and dimerization domains in gray, compared with that of bacteriophage HK97 gp5 (**e**). **f**, **g** Part of the MCP network viewed from outside **f** and inside **g** the capsid. **h**–**k** Three types of network interactions among hexon MCPs. **h** Type III interaction builds on and fortifies type I interaction. **j** Type I interaction, an intra-capsomeric augmentation of β-strands from adjacent MCPs (e.g., P2 and P3) in the same capsomer. **i** Type II interactions, inter-capsomeric interactions among a pair of MCPs (e.g., P3 and C6), join two dimerization domains. **k** Type III interactions, characterized by the lassoing action of the N-lasso domain (e.g., P3, C5, and C6), occur among three MCPs. See also Supplementary Movie 5.

The 12 penton and 150 hexon capsomers are each composed of five and six pairs of MCP and SCP, respectively. The structure of the 1345 amino-acid (a.a.) long HHV-6B MCP monomer of each capsomer subunit is divided into “tower” and “floor” regions based on their spatial positions relative to the virion capsid shell. The “tower” region contains the upper (a.a. 479–1022), the channel (a.a. 397–478 and 1296–1345), the buttress (a.a. 1086–1295), and the helix-hairpin (a.a. 189–231) domains; the “floor” region contains a dimerization (a.a. 290–361), the N-lasso (a.a. 1–58), and the bacteriophage HK97-like, “Johnson-fold” (a.a. 59–188, 232–289, 362–396, and 1023–1085) domains (Fig. 2a). The Johnson-fold domain is named after a characteristic fold first identified in bacteriophage HK97 gp5 (ref. ^32^) (Fig. 2e), which was later found in the major capsid proteins of many DNA bacteriophages^33,34^ and herpesviruses^4,5,7–9^. As shown in Fig. 2d, the Johnson-fold domain in HHV-6B MCP serves as an organizational hub, responsible for the insertion or attachment of the other six MCP domains. Though MCP of HHV-6B is 26 residues shorter than that of HCMV, there is no recognizable insertion of sequence segments. Their structures are nearly identical throughout all the seven domains, with only noticeable differences among loops at the tip of the upper domain and the loop-rich region of the buttress domain (Supplementary Fig. 9a, b).

First recognized in HCMV^8^ and subsequently confirmed in HSV^5–7^ and KSHV^4^, three types of MCP–MCP interactions within the floor region are key to establishing the HHV-6B capsid shell (Fig. 2f–k and Supplementary Movie 5). Type I interaction is intra-capsomeric, formed between two adjacent MCPs. P2 and P3 MCPs exemplify this type: two β-strands in the N-lasso of P2 MCP, two β-strands in the E-loop of the Johnson-fold, and one β-strand in the dimerization domain of P3 MCP integrate into a five-stranded β-sheet (Fig. 2j, from outside the capsid view). Type II interaction is inter-capsomeric, formed between two MCPs belonging neighboring hexons, such as P3 and C6 MCPs (Fig. 2i, from inside the capsid view). Type III interaction exists among three MCPs and is distinguished by the lassoing action of the N-lasso domain (highlighted by blue, green, and brown in Fig. 2k). In addition, type III interaction builds upon and likely consolidate type I interaction, as exemplified in Fig. 2h, by extending the N-lasso of C5 MCP to lash around β-strands in the N-lasso of P2 MCP and E-loop of P3 MCP.

Among all HHV-6B capsid proteins, the U32 gene product SCP is the smallest. Our SCP model encompasses residues 23–83 of the 89 residue-long SCP and consists of three α-helices and two connecting loops, folded into a triangular spiral with the N-terminal helix (H1) pointing outwards (Fig. 2b). For all SCP copies in our density maps, the density quality for residues 1–22 gradually degrades from highly disordered to completely invisible toward the N terminus, suggesting that this N-terminal fragment is inherently more flexible than the rest of the protein and thus was not as well resolved in the cryoEM maps obtained by averaging thousands of individual viral particles. Our model shows that SCP’s helix H3 contributes the largest number of interacting residues between SCP and MCP, inserting itself into a groove in the upper domain of MCP (Fig. 2b). SCPs in HHV-6B bind both penton and hexon MCPs and each MCP is bound by exactly one SCP (Fig. 2c).

### Heterotrimeric triplex seals the hole amid three capsomers

**Fig. 3:**
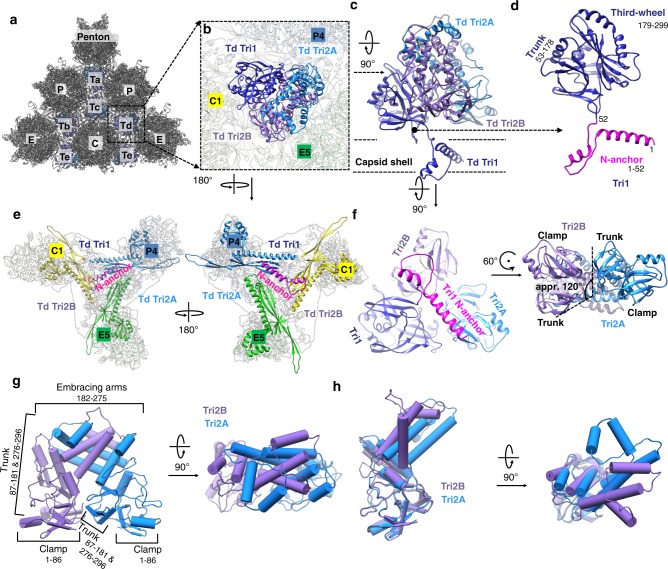
Structure of triplex and the functional significance of Tri1 N-anchor. **a** Distribution of triplexes Ta, Tb, Tc, Td, and Te among three types of hexons (C, E, and P) and a penton. **b** Enlarged view of a triplex Td with three adjacent hexon MCP subunits labeled (C1, E5, and P4). MCP subunits have been faded. **c** Further enhanced structure of triplex Td, rotated 90 degrees clockwise on the *x* axis from **b**. **d** Model of Tri1 with labeled domains. **e** Top and bottom views of **b**, showing that triplex Td is anchored to the capsid floor by the “V”-shaped Tri1 N-anchor (in pink). **f** Triplex Td viewed from inside of the capsid (left), counterclockwise rotation of Tri2A by 120 degrees about a local threefold axis results in a superposition of clamp and trunk domains from Tri2A to those from Tri2B, suggesting a high level of similarity (right). **g** Pipe-and-plank representations of Tri2 dimer in side (left panel) and top (right panel) views showing the helix bundle formed from Tri2A and Tri2B’s embracing arm domains. **h** Two orthogonal views of superposition of Tri2A and Tri2B showing nearly identical clamp and trunk domains, but different embracing arms. See also Supplementary Movie 6.

Each of the above-mentioned 320 triplexes is a heterotrimer (Fig. 3a–c) that consists of one unique conformer of Tri1 (Fig. 3d) and two Tri2 conformers (Tri2A and Tri2B) that “embrace” each other to form a dimer (Fig. 3g). The Tri1 monomer attaches to the side of the two “embracing” Tri2 subunits (Fig. 3c), like a “third wheel” (Supplementary Movie 6).

Tri1 consists of three domains: N-anchor (a.a. 1–52), trunk (a.a. 53–178), and “third-wheel” (a.a. 179–299) (Fig. 1f and Fig. 3d). The helix-loop-helix-loop motif of the N-anchor domain penetrates the capsid shell near its local threefold axis (Fig. 3c), such that the two helices fill two of the three MCP valleys (Fig. 3e and Supplementary Movie 1). The valley underneath the MCP floor is formed between the P-subdomain β-sheet and the spine helix of the Johnson-fold domain of each MCP. Through this structure, the N-anchor *anchors* Tri1 and the entire triplex from inside the capsid and beneath the MCP floor region, simultaneously sealing the hole at the local threefold axis where three neighboring MCP P-subdomains encounter one another. Notably, such “internally anchored” interactions could be “pressure-fortified”^5^, that is, when the N-anchors are pressed against the MCP floor region within the capsid by incoming DNA during genome packaging, the capsid floor would be better sealed and further strengthened rather than weakened. The N-anchor structure of HHV-6B Tri1 differs from those in other known herpesvirus Tri1 structures, including that of HCMV Tri1 (Supplementary Fig. 9c, d).

Tri2 consists of three domains: clamp (a.a. 1–86), trunk (a.a. 87–181 and 276–296), and an embracing arm (a.a. 182–275) (Fig. 1f and Fig. 3g). The two conformers of Tri2 in each triplex—Tri2A and Tri2B—differ in structures; in particular, their embracing arm domains are rotated ~ 45-degrees from each other (Fig. 3h). By contrast, their trunk and clamp domains are highly similar (Fig. 3h) and can be related to one another by a rotation of 120 degrees when viewed from the top (Fig. 3f).

### Tegument protein pU11 and its interaction interfaces

**Fig. 4:**
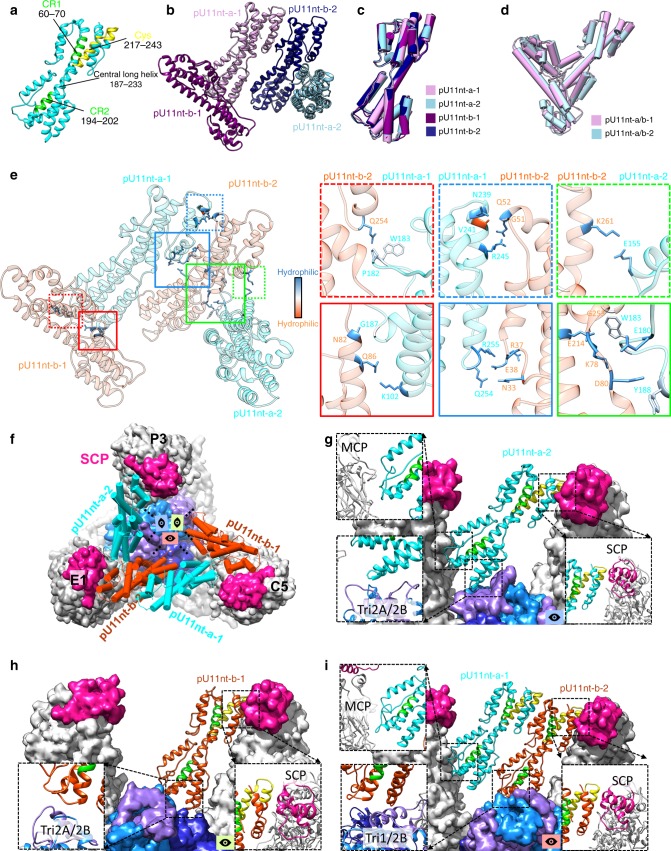
pU11 forms tetrameric CATC and CATC-capsid interactions. **a** Atomic model of pU11nt, shown as ribbon. Green residues color β-herpesvirus-conserved regions CR1 and CR2, whereas yellow residues color the cys region, conserved from primate CMV, in pU11nt. **b** Structure of a pU11nt tetramer in HHV-6B. **c**, **d** Structural alignment, based on Cα atoms, of the four pU11nt monomers (**c**) and two pU11nt dimers (**d**) from triplex Tb region reveals high levels of structural similarity. **e** Residues involved in HHV-6B pU11nt-pU11nt interactions (i.e., whose atoms are within 3.2 Å from each other, exemplified here by the triplex Te region) are solid colored with hydrophobicity and hydrophilicity depicted and shown in enlarged boxes in the right panels. pU11nt-a-1 has nine residue interactions, four with pU11nt-b-1 (red dash and solid boxes) and five with pU11nt-b-2 (blue dash and solid boxes), whereas pU11nt-a-2 has four residue interactions with pU11nt-b-2 (green dash and solid boxes). **f** Top–down view along a local threefold axis of the triplex Td region. A pU11nt tetramer (pU11nt-a-1/2, cyan, and pU11nt-b-1/2, orange red) binds to the triplex and extends towards the SCPs (magenta) that lie atop nearby MCPs (gray). **g**–**i** Side views of the structure in **f**, showing how pU11nt-a-2 (**g**), pU11nt-b-1 (**h**), and pU11nt-a-1/pU11nt-b-2 (**i**) interact with capsid proteins. The interaction interfaces are shown in enlarged boxes. See also Supplementary Movie 7.

Our atomic model of HHV-6B pU11 only contains the N-terminal one-third (pU11nt, a.a.7–275) of the 858 a.a.-long pU11; the remaining part of the C-terminal two-thirds is disordered and invisible in our density map. pU11nt is dominated by α-helices (Fig. 4a) and characterized by upper and lower helix bundles joined by a long central helix (~ 68 Å in length, a.a. 187–233) (Fig. 1f and Fig. 4a). This structure differs from that of pUL32/pp150nt of HCMV mainly in their lower domain (Supplementary Fig. 10c, d). We also identified conserved regions 1 (CR1) and 2 (CR2) in HHV-6B pU11nt (Fig. 4a); however, these regions do not contain any cys tetrad identified in the corresponding sequence regions in HCMV pUL32 (Supplementary Fig. 11)^35^. Atop the triplex, four pU11nt subunits cluster into a tetramer (Fig. 1c–e) in a “VΛ”-shaped, dimer-of-dimers configuration and their subunits can be classified into four types based on their relative locations in the dimer-of-dimers: pU11nt-a-1, pU11nt-a-2, pU11nt-b-1, and pU11nt-b-2 (Fig. 4b). These four pU11nt subunits share similar structures (Fig. 4c) with an RMSD of 0.79 Å among them. The level of similarity is even higher when the two dimers are superposed (Fig. 4d) with an RMSD of only 0.57 Å. As shown in Fig. 4e, just a couple of side-chain interactions are involved in the **a** and **b** conformers of each dimer and between the two dimers, and these interactions bear no similarities to one another.

Figure 4f–i and Supplementary Movie 7 show how a pU11nt tetramer interacts with capsid proteins that rationalizes the binding of as many as four pU11nt subunits atop each triplex Tb, Td, or Te in HHV-6B. The subunits of the pU11nt tetramers cluster on each triplex and lean against three neighboring pairs of MCP and SCP (Fig. 4f). Although the four pU11nt subunits have highly similar structures, their interactions with neighboring capsid proteins differ, exhibiting remarkable multi-specificities in these interactions. First, the upper domain of pU11nt interacts with both SCP (Fig. 4g–i) and the upper domain of another pU11nt (Fig. 4i). Second, the lower domain of pU11nt-b-1 interacts with Tri2A and Tri2B (Fig. 4h), whereas that of pU11nt-b-2 interacts with Tri1 and Tri2B (Fig. 4i). Third, even though both pU11nt-a-1 and pU11nt-a-2 have similar interactions with an MCP (Fig. 4g, i), only the lower domain of pU11nt-a-2 interacts with Tri2A and Tri2B (Fig. 4g); by contrast, that of pU11nt-a-1 interacts with the lower domain of the pU11nt-b-2 (Fig. 4i).

### Capsid binding by HHV-6B pU11, MCMV pM32, and HCMV pUL32

**Fig. 5:**
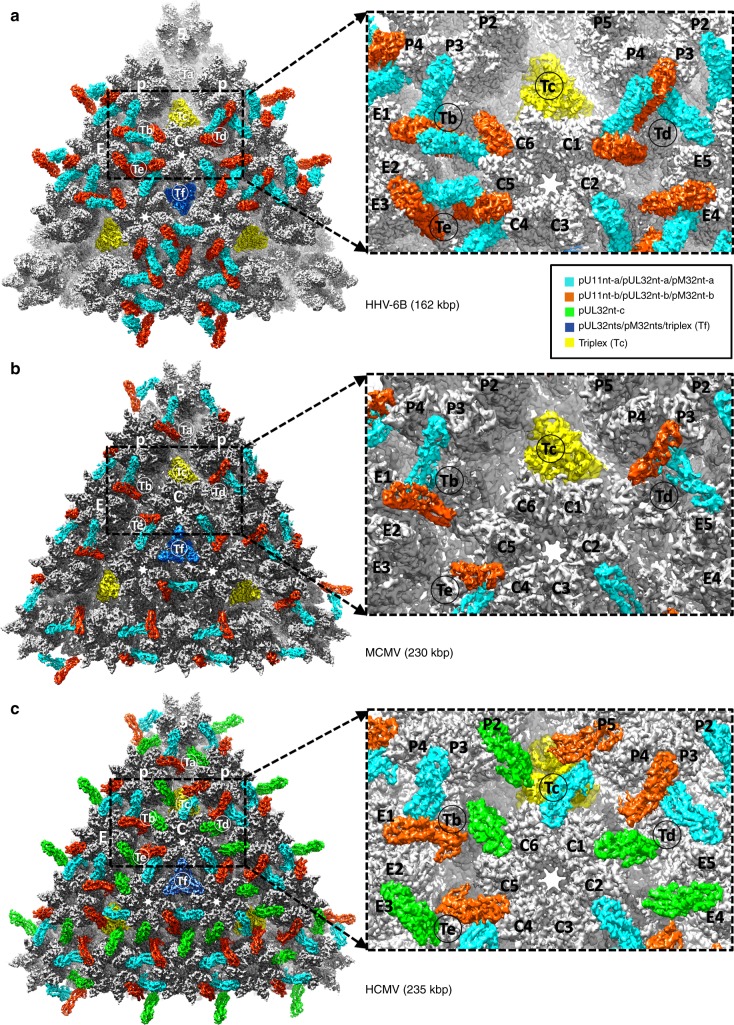
Comparison of CATC-capsid-binding patterns in HHV-6B, MCMV, and HCMV. **a** A triangular facet of the HHV-6B icosahedral reconstruction. Four pU11nt subunits form a dimer-of-dimers, “VΛ”-shaped tetramer (cyan and orange red) atop triplexes Tb, Td, and Te, but absent from triplexes Ta, Tc (yellow), and Tf (blue). **b** The corresponding triangular facet in an MCMV icosahedral reconstruction^9^. Except for triplex Tc (yellow), two pM32nt subunits form a “Λ”-shaped dimer density (cyan and orange), with each of the two arms of the “Λ” holding to a neighboring hexon/penton capsomere like the cables of a cable-stayed bridge, and its vertex of the “Λ” sitting atop a triplex. **c** The corresponding triangular facet of an HCMV icosahedral reconstruction^8^. Three pUL32nt subunits form a “Δ”-shaped CATC densities atop every triplex. In all three panels, the density of pM32nt/pUL32nt and triplex in Tf region were colored in blue, as the densities in this region were smeared after imposing threefold symmetry during icosahedral reconstruction.

Although HHV-6B, MCMV, and HCMV all are members of the β-herpesvirus subfamily, our HHV-6B reconstruction reveals that its teguments and capsomers associate in manners strikingly different from those found in MCMV and HCMV (Fig. 5a–c). Their differences can be considered from three respects.

First, the HHV-6B reconstruction shows 180 pU11-related CATC densities (Fig. 5a), as opposed to the 255 pM32-related CATC densities in MCMV (Fig. 5b) and the 310 pUL32 related CATC densities in HCMV (Fig. 5c) (note: though the icosahedral reconstructions shows 260 and 320 densities owing to symmetrization, one of the 12 vertices is the portal vertex and the sites above peri-portal triplexes Ta and Tc are occupied by the pentameric helix-bundling CVSC complexes, leading to 255 and 310 pM32/pUL32 CATC densities/capsid in each MCMV and HCMV, respectively; see Discussion)^8,9,36^. CATC exists as a group-of-four subunits in HHV-6B (Fig. 5a), but as a group-of-two subunits in MCMV (Fig. 5b) and a group-of-three subunits in HCMV (Fig. 5c); it is arranged in a “VΛ” formation in HHV-6B, “Λ” in MCMV, and “Δ” in HCMV.

Second, as shown in Fig. 5a, pU11 only binds to triplexes Tb, Td, and Te in the HHV-6B capsid. In contrast, pM32 binds to all but triplex Tc in MCMV (Fig. 5b), and pUL32 binds to all triplexes in HCMV (Fig. 5c). pU11 density connections in HHV-6B are observed between edge and facet capsomers. Specifically, pU11 tetramer of the edge type (pp150 on triplexes Ta, Tb, and Td) joins P hexons, E hexons, and C hexons (Fig. 1c and Fig. 5a)—but not pentons—on the 30 edges of the icosahedral capsid to triplexes Tb and Td, whereas pU11 tetramers of the facet type (pp150 on triplexes Tc, Te, and Tf) binds three C hexons together in the center of each icosahedral facet to triplex Te (Fig. 1c and Fig. 5a).

Third, among the four pU11 subunits within each CATC tetramer in HHV-6B, three (pU11nt-b-1, pU11nt-b-2, and pU11nt-a-2) exhibit organizational similarity to the three pUL32 subunits bound to each triplex in HCMV, though their relative inter-subunit translation and rotation differ slightly (Supplementary Fig. 12a). Likewise, the pU11nt-2 dimer in HHV-6B resembles the dimer of triplex-bound pM32 in MCMV, with the relative inter-subunit orientation within this dimer distinct (Supplementary Fig. 12b).

As described above, no pU11 binds to triplexes Ta, Tc, and Tf in HHV-6B (Fig. 5a). Rigid-body fitting of triplex Tb with its associated pU11nt tetramer into the triplex Ta density (Supplementary Fig. 13a, b) reveals that the distance between pU11nt-b-2′s upper domain to the adjacent SCPs is greater at triplex Ta than that at triplex Tb (13 Å vs. 6 Å) (Supplementary Fig. 13d). In addition, the upper domain of the superposed pU11nt-b-1 clashes substantially with the neighboring SCP (Supplementary Fig. 13c). Conceivably, the rigidity of the helix-rich pU11nt tetramer may have made it impossible to extend in order to span a longer distance, or to shrink in order to prevent clashes, thus explaining the absence of pU11 above triplex Ta in HHV-6B. Likewise, the rigidity of pU11 tetramer is incompatible with its binding to triplexes Tc and Tf, either owing to too long a distance to reach the adjacent SCP or too short a distance to clash into the neighboring SCP (Supplementary Fig. 13e–j).

## Discussion

By using cutting-edge cryoEM imaging and sub-particle reconstruction methods, we have successfully determined the near-atomic resolution structure of HHV-6B using crude, minimally purified samples. As a result, we derived the atomic model of both the HHV-6B capsid and its capsid-associated tegument complex of pU11 tetramer. This represents the fifth-ever human herpesvirus whose capsid structure has been described at atomic level, following HCMV, herpes simplex virus 1 and 2 (HSV-1 and HSV-2), and KSHV. HHV-6B’s capsid structure is similar to known capsid structures of other herpesviruses with only minor differences (Supplementary Figs. 9–10). Like other herpesviruses, HHV-6B capsid contains three types of MCP interactions, all of which are fortified by inter-capsomeric triplexes through the Tri1 N-terminal anchor. Beyond the capsid, the helix-rich tegument protein pU11 forms tetramers, bridging the space between the upper domain of the hexon MCP and the triplexes Tb, Td, and Te. These triplexes are only three of the six triplexes at quasi-equivalent positions in each icosahedral asymmetric unit.

To date, the only known structure for HHV-6B proteins is the crystal structure of the N-terminal portion (a.a. 2–458) of pU14 (pU14nt)^11^. pU14 is a 603 amino-acid long, β-herpesvirus-specific tegument protein (pp85 superfamily) that is present in the HHV-6B virion, though its exact location within the tegument compartment is not known. pU14nt forms an elongated helix-rich fold, with a β hairpin protruding out from the body of the C-terminal region (a.a. 330–458) (Supplementary Fig. 14a–b). Surprisingly, pU11nt and pU14nt share a similar fold for the first 300 amino acids (Supplementary Fig. 14a–b). Although our cryoEM structure of pU11 was obtained in situ from the wild-type full-length protein of pU11 inside the virion, only pU11’s N-terminal one-third structure, which has an elongated helix-rich fold, is resolved; the remaining part of the C-terminal two-thirds of the protein is unstructured (Supplementary Fig. 14c), consistent with sequence-based structure prediction (Supplementary Fig. 11). By contrast, a.a. 330–455 of pU14 forms a well-structured globular domain, with a characteristic β-hairpin protrusion (Supplementary Fig. 14b).

In the previously determined crystal structure of the N-terminal portion (a.a. 2–458) of pU14 (pU14nt), two pU14nts form a dimer in an anti-parallel “handshake” orientation, with the protruding β hairpins positioned like thumbs in a handshake. This anti-parallel orientation is in stark contrast to the vertical inter-subunit orientation seen in the pU11 tetramer in our cryoEM structure. The β-hairpin protrusion of the additional structured domain from a.a. 330–455 of pU14 has an important role in the handshake dimer arrangement. Unlike pU11, which primarily has a structural role in securing capsid integrity (discussed above), pU14 appears to be a “cargo” protein packaged into the tegument compartment of the virion, serving functional roles for viral infection. Such roles include viral propagation^37^ and interacting with host factors such as tumor suppressor p53^38^ and cellular protein EDD^39^ to regulate host cell responses. This fold similarity between pU11 and pU14 suggests that these two β-herpesvirus-specific tegument proteins might share a common ancestry with the helix-rich fold. Gene duplication might have co-opted this fold to append novel C-terminal sequences, creating vastly different oligomeric forms to serve different structural and functional roles.

HCMV protein pp150/pUL32, the tegument protein homologous to HHV-6B pU11 and MCMV pM32, has been known to be highly phosphorylated and multi-functional. Previous studies have suggested that tegument phosphoproteins such as pp150 are responsible for stabilizing cytoplasmic capsids and controlling their movements, especially in β-herpesviruses that experience higher internal pressure from their larger genome sizes^40,41^. In HCMV, pp150nt forms helix bundles, securing the capsid through a cysteine triad and effectively stabilizing the HCMV capsid^8^. MCMV similarly contains numerous stabilizing pM32 dimers; however, deletion mutagenesis suggests that its role is only limited^9^. In HCMV, MCMV, and HHV-6B, the number of tegument phosphoprotein subunits decreases with the genome sizes of the respective viruses, with HCMV containing the largest number of subunits, followed by MCMV and then HHV-6B. This observation is in line with a role of pp150 or its homologs to secure the pressurized capsid after encapsidating a dsDNA genome^9,42^. Given that the genome size (162 kb) of HHV-6B is just slightly larger than those (~ 150 kb) of α- and γ-herpesviruses, it is conceivable that the need for pU11 to stabilize a pressurized genome-containing capsid is much diminished, if any. Rather, pU11 may have alternative roles in HHV-6B assembly and infection, as expected also for pp150 in HCMV^12,43^.

Among the several CATCs resolved structurally to date, the capsid-vertex specific components (CVSC)^36,44,45^ are genetically conserved among subfamilies of herpesviruses, in contrast to the pU11-related pp150 CATC, which are specific to β-herpesviruses. CVSC have been proposed to be vital for viral propagation and DNA packaging^46^. CVSC has been observed in the past to bind to triplex Ta and Tc in herpesvirus capsids^5,47^. In α-herpesviruses HSV-1 (ref. ^5^), HSV-2 (ref. ^6^) and PrV^48^, each CVSC complex is a pentamer, consisting of two copies of pUL25, two copies of pUL36, and one copy of pUL17, each contributing a helix to form a helix bundle. Five CVSC complexes associate through their combined 10 pUL25 head domains to form a pentagram, crowning each of the 11 icosahedral vertices^5,6^, as well as the portal vertex^24,29^. Similarly, in γ-herpesvirus KSHV, five subunits—two copies of pORF19, two copies of pORF64, and one copy of pORF32—join to form a CVSC; in term, five CVSC complexes join through five of the 10 pORF19 head domains to form a pentagram crown which caps the portal vertex^18,47^. However, some pentonal vertices of KSHV, particularly those farther away from the portal vertex, have less than five CVSC complexes bound^18^. In HCMV, all prospective CVSC-binding sites on triplexes Ta and Tc near the pentonal vertexes are occupied by pUL32/pp150 tegument proteins; as such, its conserved CVSC proteins are expected to only bind to the portal vertex. In MCMV, triplex Ta was similarly occupied by pM32 and no CVSC complexes were observed on triplexes Ta and Tc.

Our atomic model reveals that binding between CVSC and the capsid of herpesviruses is not determined solely by the availability of triplex binding sites on the capsid. It is natural to expect the binding pattern of CVSC tegument proteins on pentons to be dictated by the availability of its binding partners (Ta and Tc). This pattern is indeed consistent in α-herpesviruses HSV-1, HSV-2, and PrV, where CVSCs were observed at appropriate locations, as well as in β-herpesviruses MCMV^9^ and HCMV^42^, where the lack of CVSC binding at penton vertices is expected because the corresponding pentonal CVSC-binding locations (triplex Ta and Tc) are already occupied by pM32 and pUL32 tegument proteins. Since the Ta and Tc triplexes in the HHV-6B structure were unobstructed, one would have expected to see CVSC binding on them; however, the structure shows otherwise (Fig. 5a). The lack of CVSC binding on the pentonal vertexes in HHV-6B suggests that factors other than the availability of triplexes Ta and Tc influence capsid-binding of CVSC in various herpesviruses. This raises questions regarding the nature and process of CVSC binding to the pentonal vertices of various herpesviruses-specifically, how the β-herpesvirus-specific tegument proteins influence the association of CVSC. We predict that CVSC complexes are present in HHV-6B, but only bind to the portal vertex and peri-portal triplexes Ta and Tc, rather than at the pentonal vertices and peri-pentonal triplexes Ta and Tc. Owing to the importance of CVSC in viral propagation, fully understanding the nature of CVSC binding could prove valuable towards the development of pharmaceutical drugs.

Looking forward, the significance of the atomic structure of HHV-6B presented here transcends basic knowledge about HHV-6B capsid and tegument assembly. Protein phosphorylation has been recognized as a common mechanism for regulating biological functions. Drugs against infection, inflammation, cancers, or neurodegeneration target phosphorylation pathways of human proteins; for example, searching for kinase inhibitors has been one of the major focuses of drug development during the past two decades^49–51^. Understanding the atomic structure of the phosphorylated protein pU11 in HHV-6B may prove vital to designing anti-viral drugs and vaccines against this virus.

## Methods

### Cell culture

The human T lymphoblastic cell line SupT1 (ATCC CRL-1942) was grown at 37 °C in 5% CO_2_ incubator in RPMI medium (RPMI 1640, Life Technologies), completed with 10% heat-inactivated fetal bovine serum (FBS), 1% l-Glutamine (200 nm final concentration) and 1% penicillin–streptomycin (100 UmL). Cells were seeded at a density of 0.5 × 10^6^ cells/mL and sub-cultured when density reached ~ 2–3 × 10^6^ cells/mL.

### Viral propagation and virion isolation

Cell culture and virus isolation were performed as previously described^52^. In brief, HHV-6B (Z29 strain) was propagated in SupT1 cell line. The initial inoculum contained at least 1 × 10^8^ viral copies/mL. In all, 30 mL of supernatant inoculum was thawed in a 37 °C water bath and added to uninfected SupT1 cell pellets at a concentration of 2 × 10^6^ cells/mL of inoculum. Then, the cells were plated in 5% CO_2_ incubator for 2 h and gently mixed every 30 min. At the end of the 2-hour incubation period, cells were pelleted by low-speed (~ 1800 × *g*) centrifugation for 5 min and washed three times with 1 × PBS. Cells were then resuspended in 60 mL of complete RPMI with 5% FBS at a concentration of ~ 1 × 10^6^ cells/mL. Cells were monitored closely for cytopathic effects (CPE), characterized by cytomegalic (large balloon-like cells) and syncytia (fused, multinucleated cells). In addition, viral load of the cell supernatant was monitored weekly by droplet digital PCR^53^. Healthy, uninfected SupT1 cells were added to the infected cultures when > 80% CPE was observed. Fresh completed medium with 5% of FBS was also added when the infected culture media appeared orange/yellow and in a variable volume to maintain an approximate cell density of 1–2 × 10^6^ cells/mL. Viral copies of infected supernatants were quantified prior to being aliquoted.

To minimize loss of virions and to improve structural integrity, we used a one-step isolation method to obtain crude HHV-6B materials directly for cryoEM. A total of 30 mL clarified supernatant was collected and centrifuged at 80,000 × g for 1 hr to pellet HHV-6B virions. The pellet was resuspended in 30 μL PBS overnight, evaluated for concentration and particle integrity using negative-stain transmission electron microscopy, and subsequently used for cryoEM sample preparation.

### CryoEM data acquisition

Aliquots of 2.5 μL of the above resuspended sample were applied to 200-mesh Quantifoil R2/1 grids, blotted with filter paper, and plunge-frozen in liquid ethane. CryoEM images were collected in an FEI Titan Krios cryo-electron microscope equipped with a Gatan imaging filter (GIF) on a Gatan K2 Summit direct electron detector in the super-resolution mode. Prior to imaging, the electron microscope was carefully aligned and the parallel beam was optimized using coma-free alignment in SerialEM^13^. The microscope was operated at 300 kV with the GIF slit band set at 20 eV, defocus ranges from −2.2 to −3.2 µm, and nominal magnification at × 64,000 (corresponding to a pixel size of 1.085 Å per pixel on the specimen).

Movies were recorded at a dose rate of ~ 9  e^–^ per second per physical pixel on the detector. The total exposure time for each movie was 12 s, fractionated into 60 frames with 0.2 s exposure time for each frame, leading to a total dose of 23 e^–^/Å^2^ per movie. A total of 4828 movies were collected in two sessions, each lasting ~ 70 h.

### Data pre-processing and icosahedral reconstruction

Frames in each movie were aligned and averaged to create a single micrograph by using MotionCor2 (ref. ^54^). The defocus values of these micrographs were determined with CTFFIND4 (ref. ^55^) and were found to range from −1 μm to −4 μm. A total of 7430 well-separated and artifact-free particles were picked manually and extracted into individual particle images (1280 × 1280 pixels) in Relion^27^. We binned the particle images 2 × (2.17 Å/pixel) in order to improve computation efficiency for Relion 2D classification and subsequent 3D refinement in a graphics processing unit cluster. We first classified all particles into 30 classes with Relion 2D classification to select a total of 6443 high-quality particles. We created a binary (density = 1) sphere with a radius of 640 Å and a box size of 640 Å by using a Python script and used it as the initial model to run Relion 3D refinement with icosahedral symmetry (I1). An icosahedral reconstruction at ~ 5.1 Å resolution was obtained after 55 iterations with tau_fudge of 4.

### Sub-particle reconstructions

To obtain higher resolution structures for atomic model building, we used a sub-particle reconstruction strategy^5,7,28,30^ to reconstruct sub-regions surrounding the fivefold, threefold, and twofold axes of the icosahedral HHV-6B capsid. With a script downloaded from www.opic.ox.ac.uk/localrec^28^, positions and orientations of sub-particles on the fivefold, threefold, and twofold axes in the original particle images (i.e., without binning) were calculated based on their geometric positions on the icosahedral capsid, a radial distance of 576.8 Å from the center of the viral particle and the determined icosahedral orientations with bin 4 × (4.34 Å/pixel) particles. The defocus value for each sub-particle was also recalculated based on the geometric location of the sub-particle on the capsid, thus effectively overcoming the depth-of-focus limitation encountered by icosahedral reconstruction of large particles like herpesviruses^8,31^. A total of 77,316, 128,860, and 196,290 sub-particles in 500 × 500 pixels^2^ were then boxed out with relion_preprocess for the fivefold, threefold, and twofold axis-related sub-particles, respectively. 3D reconstructions of these sub-particles were iteratively refined in Relion until convergence, reaching a final estimated resolution of 3.82 Å, 3.77 Å, and 3.77 Å for the fivefold, threefold, and twofold axis sub-particle maps, respectively, based on the 0.143 Fourier shell correlation criterion^56^ (Supplementary Table 2).

### Atomic model building and refinement

The icosahedral reconstruction of the HHV-6B particle had sufficient resolution for us to model 59 unique conformers in each AU. These conformers include 16 MCPs, 16 SCPs, 15 triplex subunits (Triplex Tf on the icosahedral threefold axis was not resolved to high resolution owing to symmetry mismatch), and 12 pU11nts. The SWISS-MODEL server^57^ was utilized to generate homology models of penton MCP, hexon MCP, SCP, Tri1, Tri2A, and Tri2B, using the subunit conformers in the atomic model of HCMV^8^ as templates (Supplementary Table 3). These initial models were docked into the sub-particle reconstructions that were sharpened with a B factor of − 120 Å^2^. For each of the 59 models, the “rotate,” “real-space refinement,” and “regularize zone” utilities in Coot^58^ were used to manually fit the residues into the density map.

These manually built models were then iteratively improved through both Phenix real-space refinement^59^ and manual readjustment in Coot. The 59 PDB files in each AU were designated into two groups: Group-One contained 42 conformers near the threefold axis, and Group-Two contained 17 conformers near the fivefold axis. All 59 conformers were subjected to three and fivefold sub-particle reconstructions independently, followed by two steps for each iteration.

In the first step, we combined all the 59 PDB coordinate files of the 59 conformers within one AU into a single AU PDB file. This AU PDB file was then used as the input model file to run real-space model refinement using the program Phenix. This program optimizes the geometry of atomic models based on threefold or fivefold sub-particle reconstructions and outputs a single AU PDB file with atomic coordinates optimized to match the cryoEM density of sub-particle reconstructions. The coordinates for the 42 subunits belonging to Group-One were refined by subjecting the AU PDB file to the real-space refinement against the threefold sub-particle reconstruction, yielding 42 refined Group-One subunits in the output AU PDB file. Likewise, the coordinates for the 17 subunits belonging to Group-Two were refined by subjecting the AU PDB file to the real-space refinement against the fivefold sub-particle reconstruction, yielding 17 refined Group-Two subunits in the output AU PDB file. The 59 molecules in each of the two output AU PDB files were then separated using a custom-designed Python script, phenixSplitter, into 59 PDB files, each containing the atomic coordinates of a monomer.

In the second step, each refined Group-One and Group-Two subunit was assessed by the wwPDB validation^60^ web server, which described the geometric outliers caused by the real space refinement during Phenix. For each PDB file, wwPDB output various outliers present (such as bond length, bond angle, planarity, and chirality), the locations of these geometric outliers, and the estimated quality of the chain. Outliers were manually fixed using the Real Space Refinement Zone, Regularize Zone, Auto-Fit Rotamer, and the Rotate Translate Zone/Chain/Molecule functions in Coot. When using the refinement tools, the molecular restraints were set up with Torsion, Planar Peptide, Trans Peptide, and Ramachandran restraints. When modifying secondary structures, the respective Mainchain Restraints were also used. Occasionally, the refinement weight was changed to accommodate the available electron density. Each corrected molecule was checked to ensure that secondary structures were not lost during real space refinement; this was done by visualizing each corrected molecule side by side with its corresponding homologous model in Coot and manually assigning secondary structures to the corrected molecule in any locations where secondary structures were lost. During each iteration, each of the refined Group-One and Group-Two subunits were manually fixed in Coot. To start the next iteration, the 42 Coot-adjusted Group-One subunits and the original 17 Group-Two subunits were combined to create one single AU file. Likewise, the 17 Coot-adjusted Group-Two subunits and the original 42 Group-One subunits were combined to create the other single AU file.

When the wwPDB report indicated that the number of outliers decreased to four and that there was no deviation in the secondary structures, we concluded that we had reached convergence for model refinement of that subunit. After all 42 Group-one subunits and 17 Group-two subunits converged, they were combined into a single AU PDB file (Supplementary Table 2).

### Reporting summary

Further information on research design is available in the Nature Research Reporting Summary linked to this article.

## Supplementary information

Supplementary Information

Description of Additional Supplementary Files

Supplementary Movie 1

Supplementary Movie 2

Supplementary Movie 3

Supplementary Movie 4

Supplementary Movie 5

Supplementary Movie 6

Supplementary Movie 7

Reporting Summary

## Data Availability

CryoEM maps and atomic models are deposited in the Electron Microscopy Data Bank (EMDB) and the RCSB Protein Data Bank (PDB), respectively. They include the cryoEM density maps of the HHV-6B capsid, sub-particle reconstructions at twofold, threefold, and fivefold axes (accession code EMD-20557, EMD-20558, EMD-20560, and EMD-20559, respectively) and a single coordinate file containing 59 atomic models (PDB accession code 6Q1F). Other data are available from the corresponding author upon reasonable request.
