# Supplementary material for: Atomic structure of the human herpesvirus 6B capsid and capsid-associated tegument complexes
Source: Nat Commun. 2019 Nov 25;10:5346. doi: 10.1038/s41467-019-13064-x (PMC6877594; doi:10.1038/s41467-019-13064-x)
Supplement: Supplementary file 1 — Supplementary Information [file 41467_2019_13064_MOESM1_ESM.pdf]

Supporting Information

Atomic structure of the Human Herpesvirus 6B Capsid and Capsid-Associated Tegument Complexes

Yibo Zhang, Wei Liu et al.

List of Content

Supplementary Figures 1-14
Supplementary Table 1-3
Supplementary References

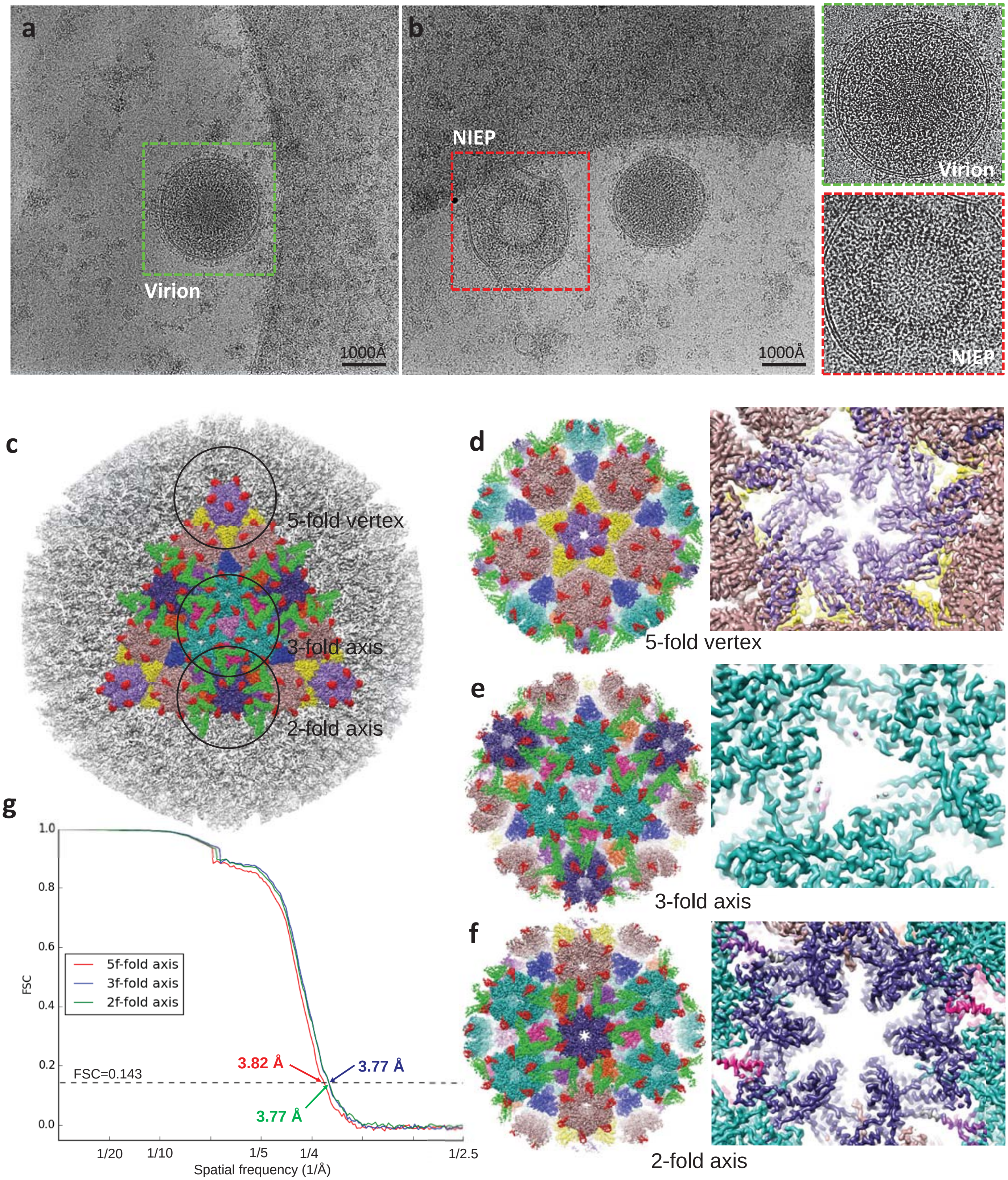

Supplementary Figure 1. CryoEM images of HHV-6B particles, sub-particle reconstructions, and resolution assessment.
(**a-b**) Representative images recorded on K2 camera indicate a low concentration of HHV-6B sample and show the HHV-6B virion (green) and non-infectious enveloped particle (NIEP, red). (**c-f**) The icosahedral reconstruction in Fig 1b is reshown (c) with one triangular facet in color; the regions (referred to as "sub-particles") surrounding a 5-fold, 3-fold, and 2-fold axis are circled. A total of 77,316 5-fold sub-particles, 128,860 3-fold sub-particles, and 196,290 2-fold sub-particles were boxed out from original particle images and refined, yielding improved resolutions for the 5-fold sub-particle (d), 3-fold sub-particle (e), and 2-fold sub-particle (f). The enlarged views from inside these sub-particle reconstructions (right panels) show α-helices and β-strands with well-resolved side chain densities. (**g**) Gold-standard Fourier shell correlation (FSC) curves of the sub-particle reconstructions, indicating the resolutions of sub-particle reconstructions at the 5-fold (red), 3-fold (blue), and 2-fold (green) axes are 3.82 Å, 3.77 Å, and 3.77 Å, respectively, based on the 0.143 FSC criterion.

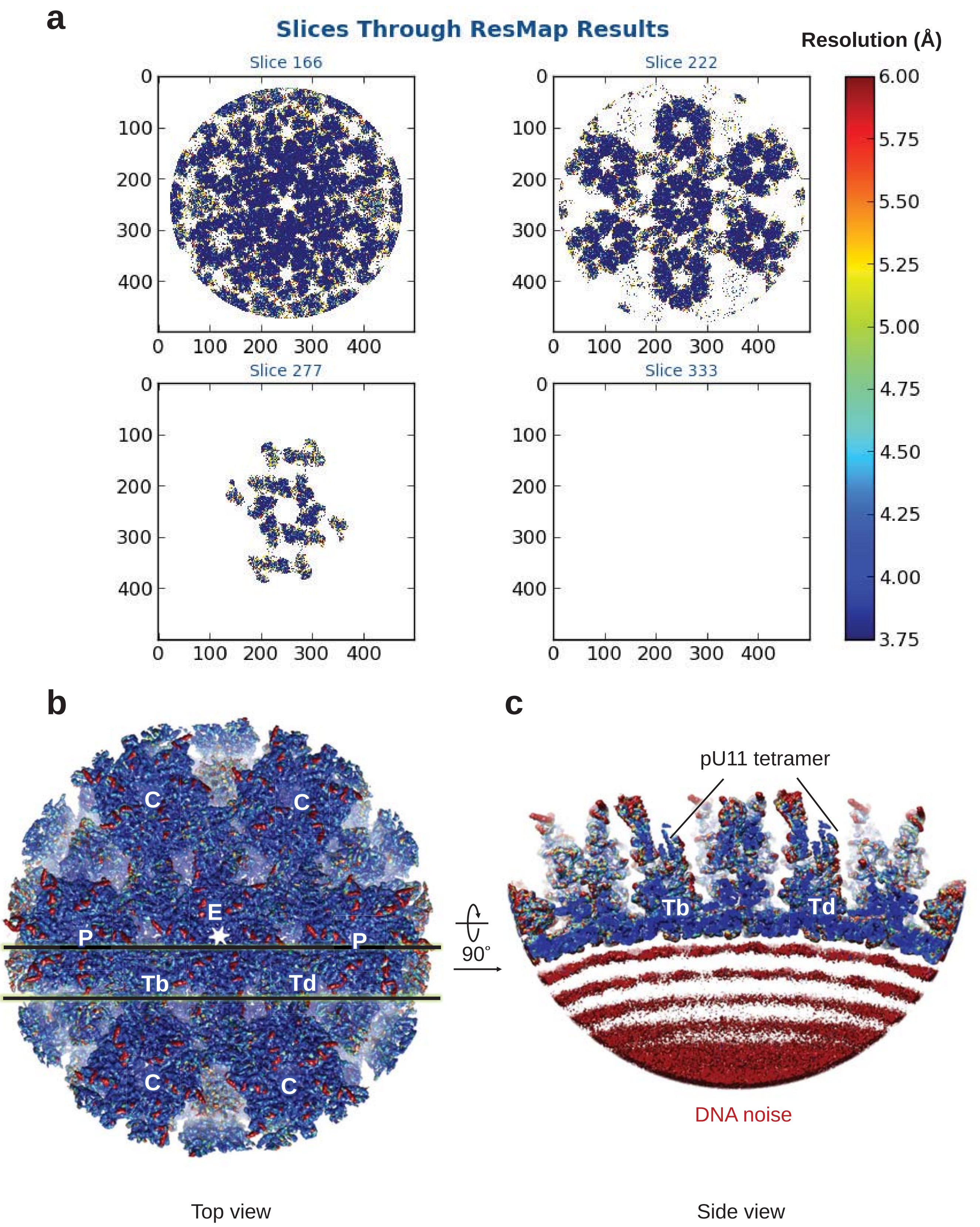

Supplementary Figure 2. Local resolution assessment for the 2-fold sub-particle reconstruction. Representative slices (**a**) and surface views (**b, c**) of the sub-particle reconstruction showing local resolution heat maps generated by *ResMap*[1]. The side view (c) shows the density slab demarked by the two horizontal lines in the top view (b). Color scheme for local resolutions is shown in the color bar. Hexons C, E, P and triplexes Tb, Td are labeled.

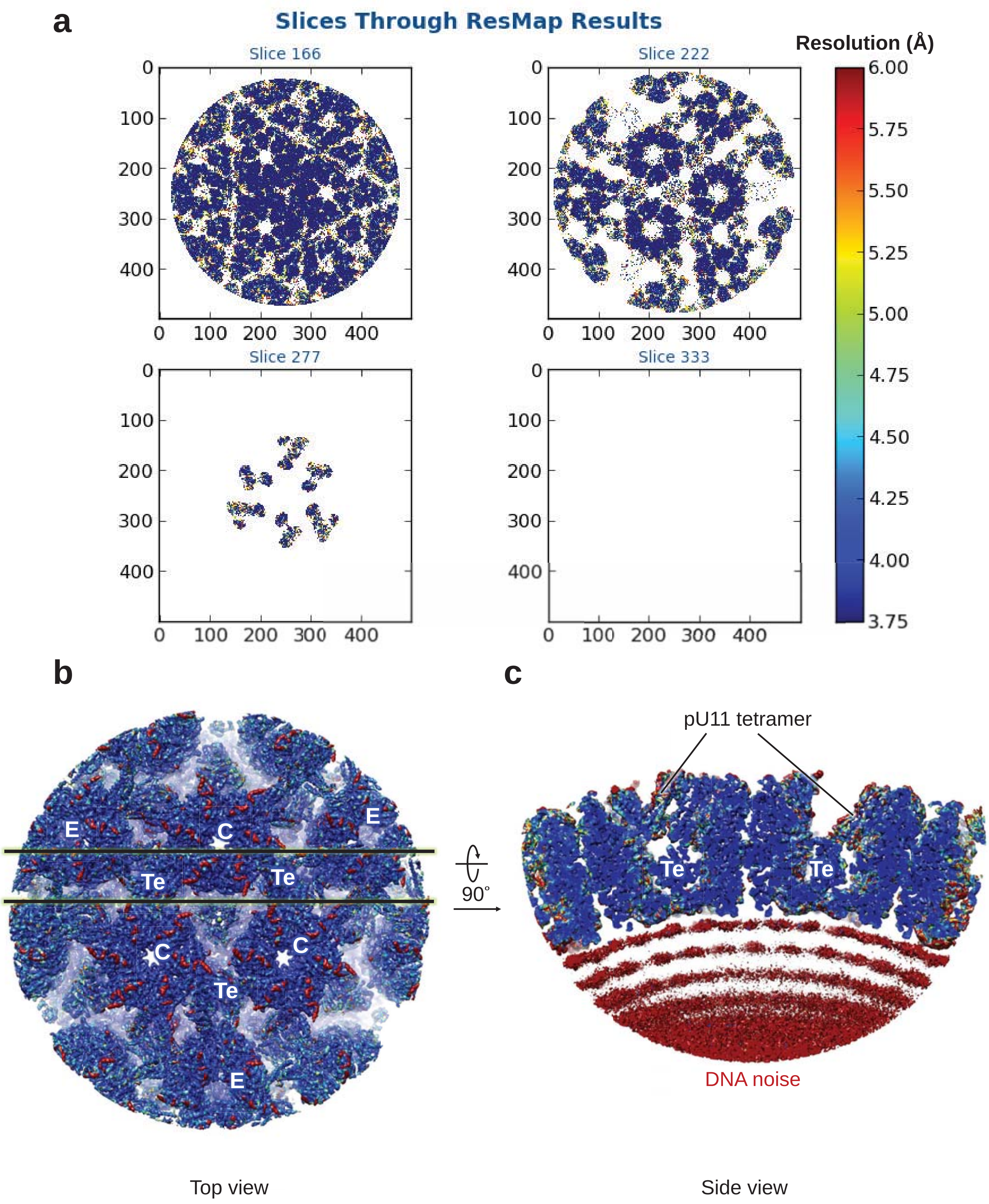

Supplementary Figure 3. Local resolution assessment for the 3-fold sub-particle reconstruction. Representative slices (**a**) and surface views (**b, c**) of the sub-particle reconstruction showing local resolution heat maps generated by *ResMap*[1]. The side view (c) shows the density slab demarked by the two horizontal lines in the top view (b). Color scheme for local resolutions is shown in the color bar. Hexons C, E and triplexes Te are labeled.

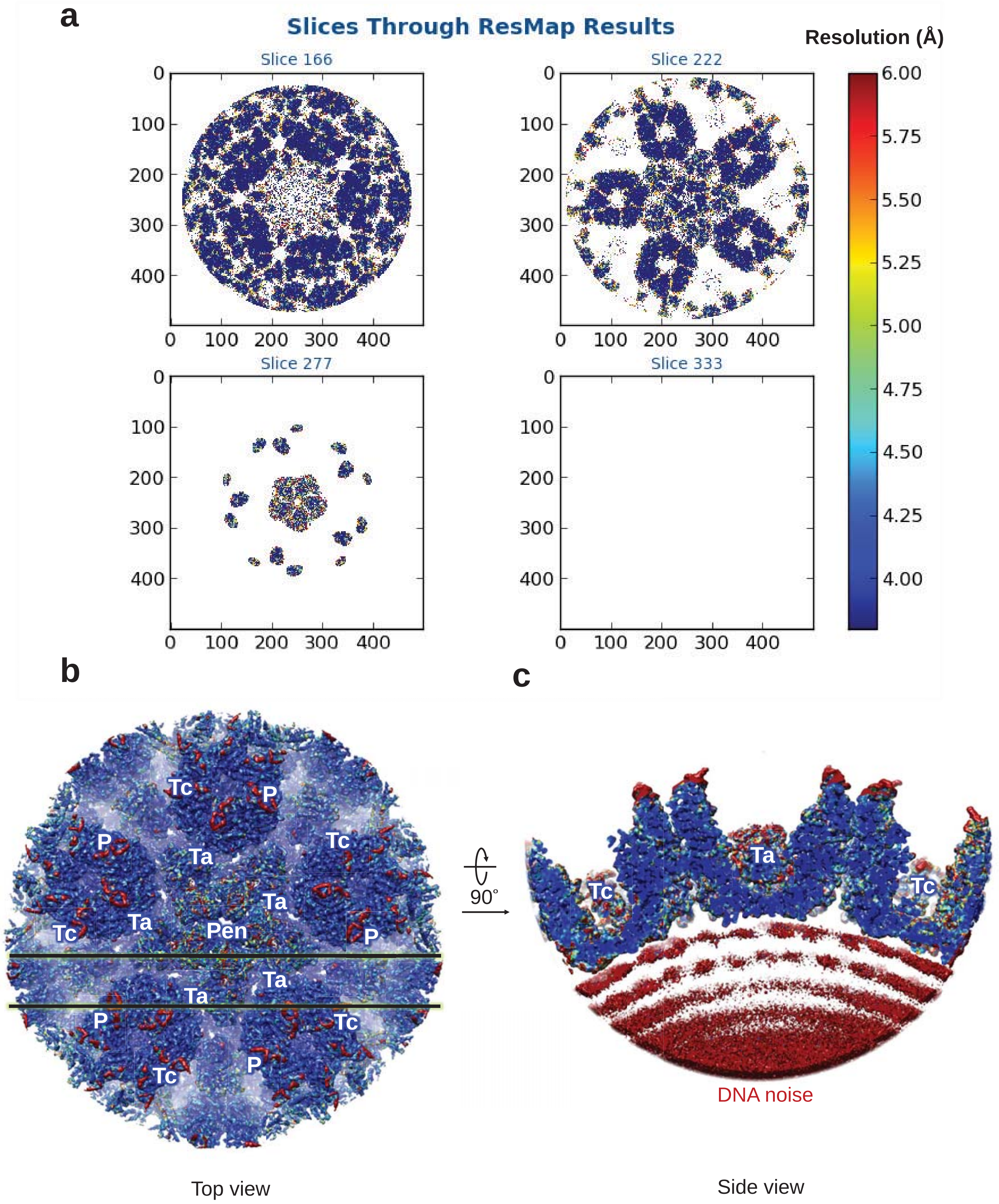

Supplementary Figure 4. Local resolution assessment for the 5-fold sub-particle reconstruction. Representative slices (**a**) and surface views (**b, c**) of the sub-particle reconstruction, showing local resolution heat maps generated by *ResMap*[1]. The side view (c) shows the density slab demarked by the two horizontal lines in the top view (b). Color scheme for local resolutions is shown in the color bar. Penton, hexons P, and triplexes Ta, Tc are labeled.

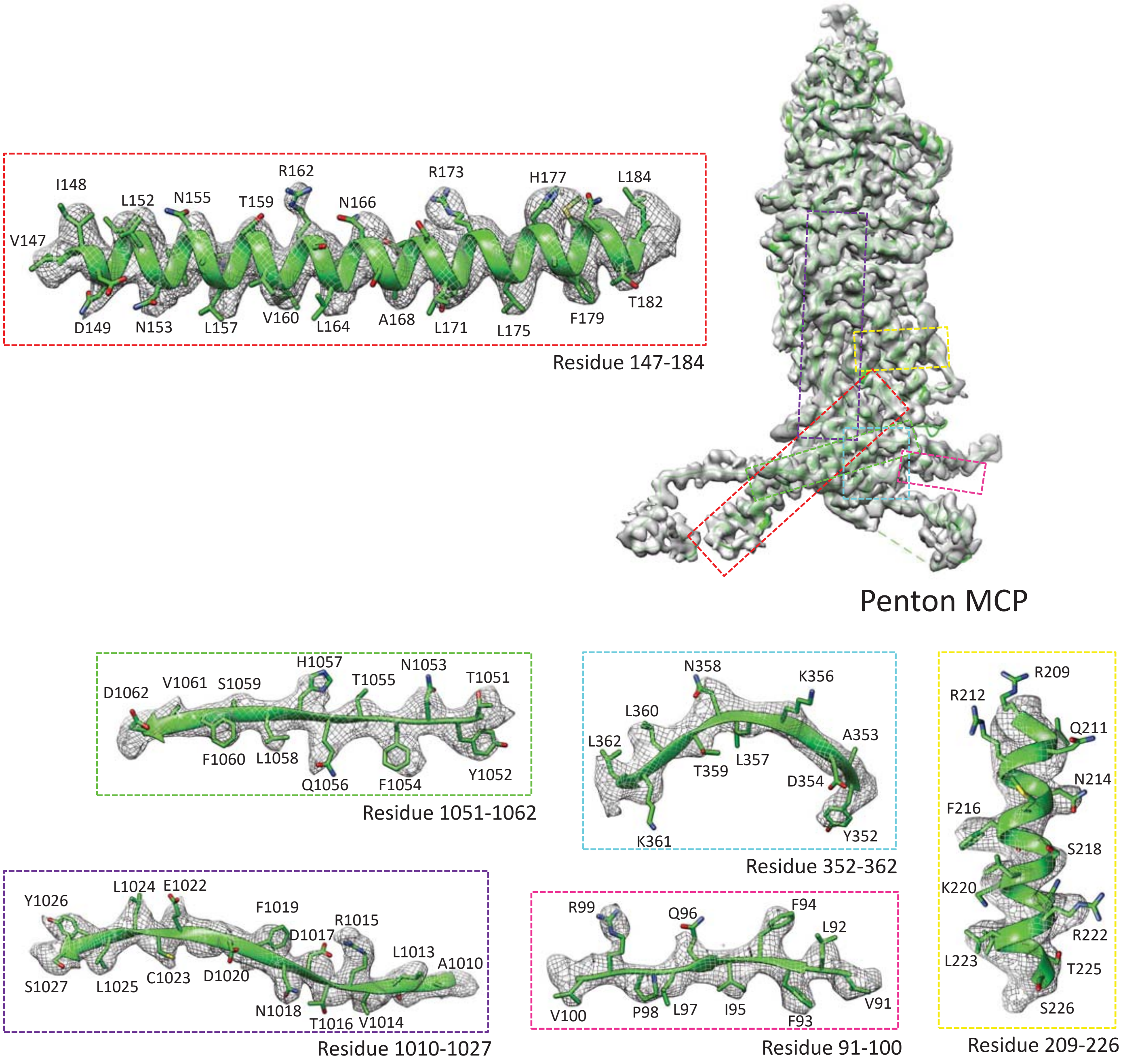

Supplementary Figure 5. Density map and atomic model of a Penton MCP.
The density map (gray) of a penton MCP (segmented out from the 5-fold axis sub-particle reconstruction) at 3.82 Å resolution is superposed with its atomic model (ribbon). Boxed regions are enlarged, with density shown as gray mesh and atomic models as ribbon/sticks, in boxes with corresponding color edges.

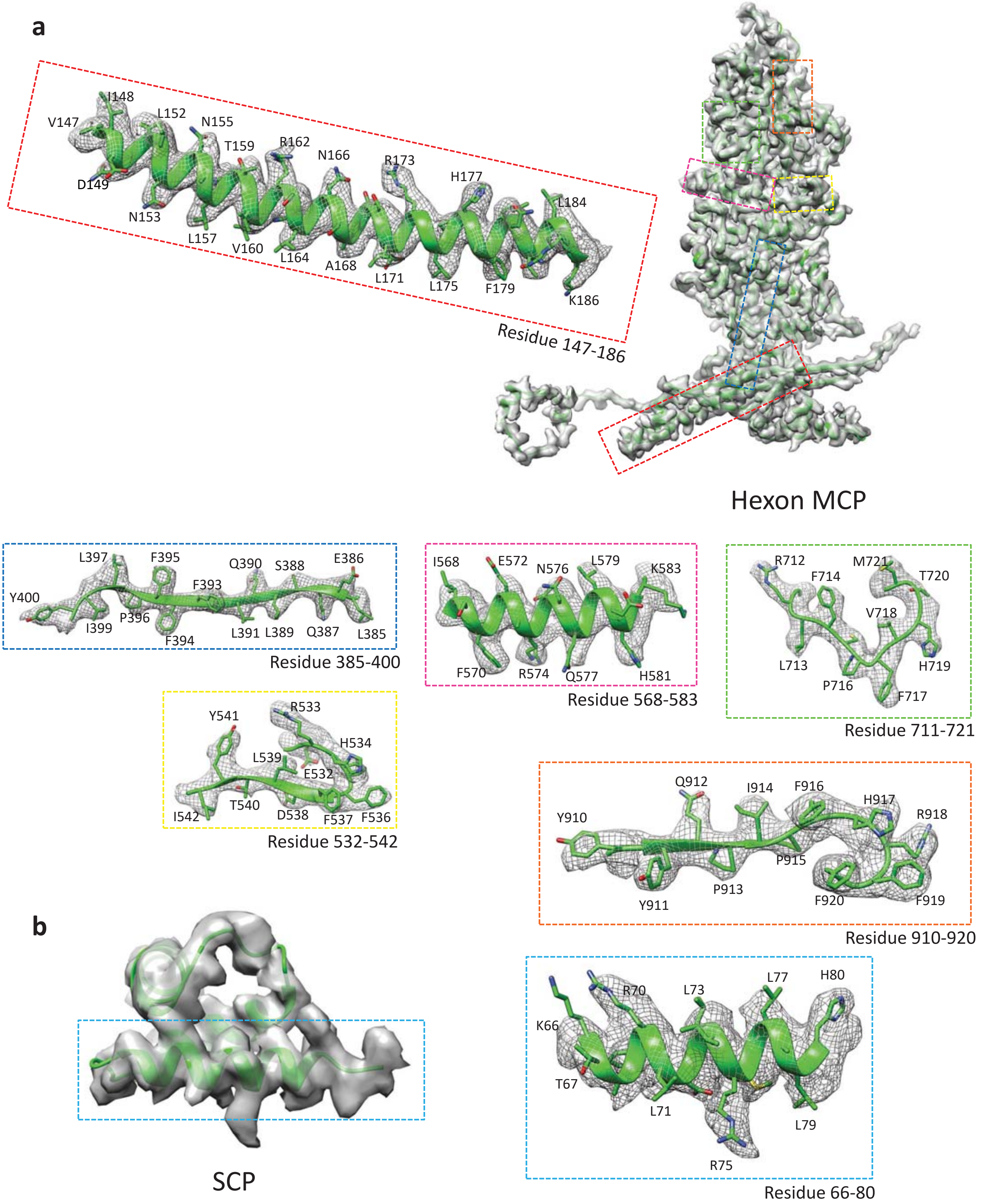

Supplementary Figure 6. Density map and atomic model of a hexon MCP and a SCP monomer.
(**a-b**) The density map (gray) of a hexon MCP (*e.g.*, MCP E2) (a) and a SCP (*e.g.*, SCP C6) (b) (segmented out from the 3-fold axis sub-particle reconstruction) at 3.77 Å resolution is superposed with the atomic model (ribbon). Boxed regions are enlarged, with density shown as gray mesh and atomic models as ribbon/sticks, in boxes with corresponding color edges.

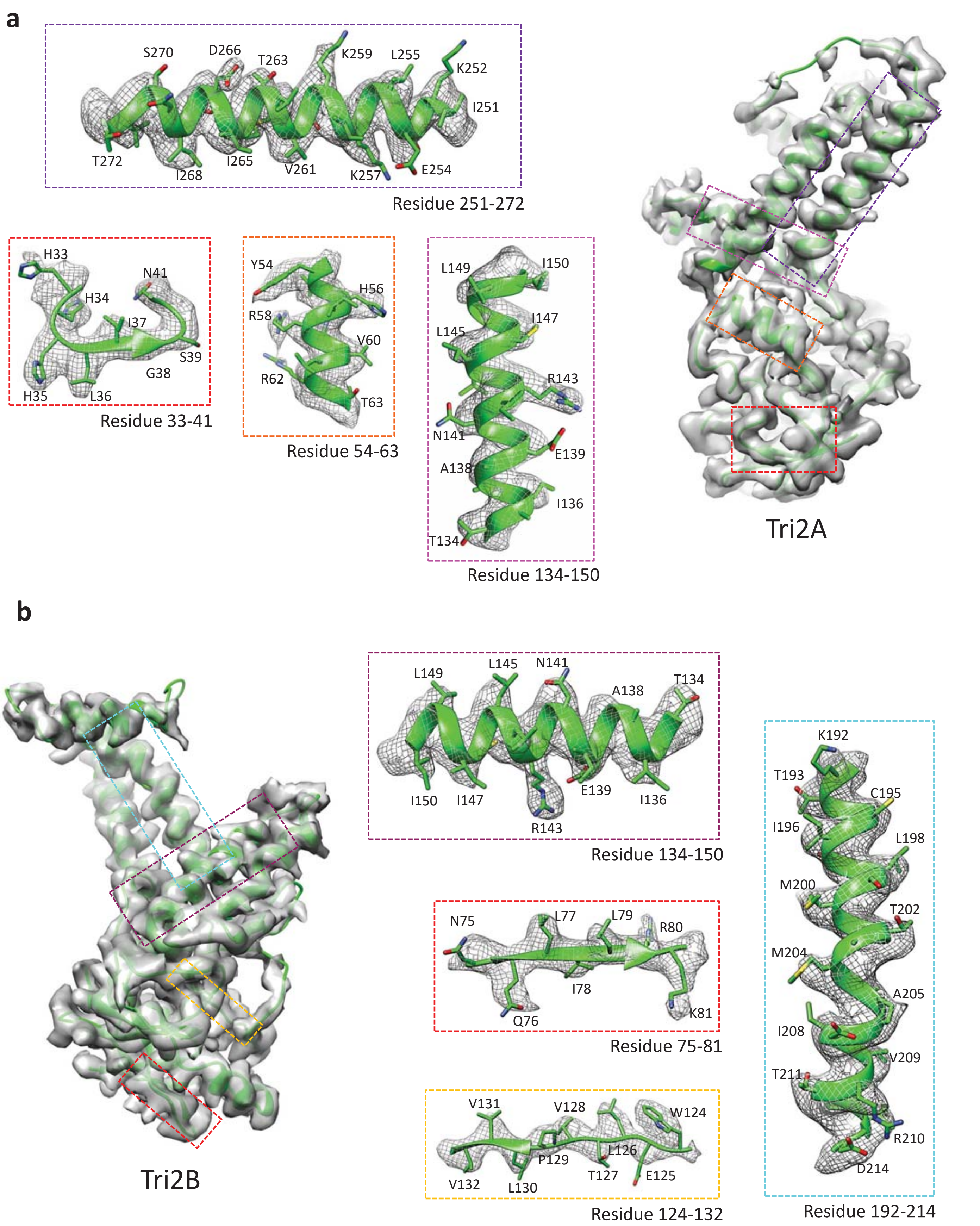

Supplementary Figure 7. Density maps and atomic models of a Tri2A and a Tri2B monomer.
(**a-b**) The density maps (gray) of a Tri2A (a) and a Tri2B (b) (segmented out from triplex Tb of the 3-fold axis sub-particle reconstruction) at 3.77 Å are superposed with their atomic models (ribbon). Boxed regions are enlarged, with density shown as gray mesh and atomic models as ribbon/sticks, in boxes with corresponding color edges.

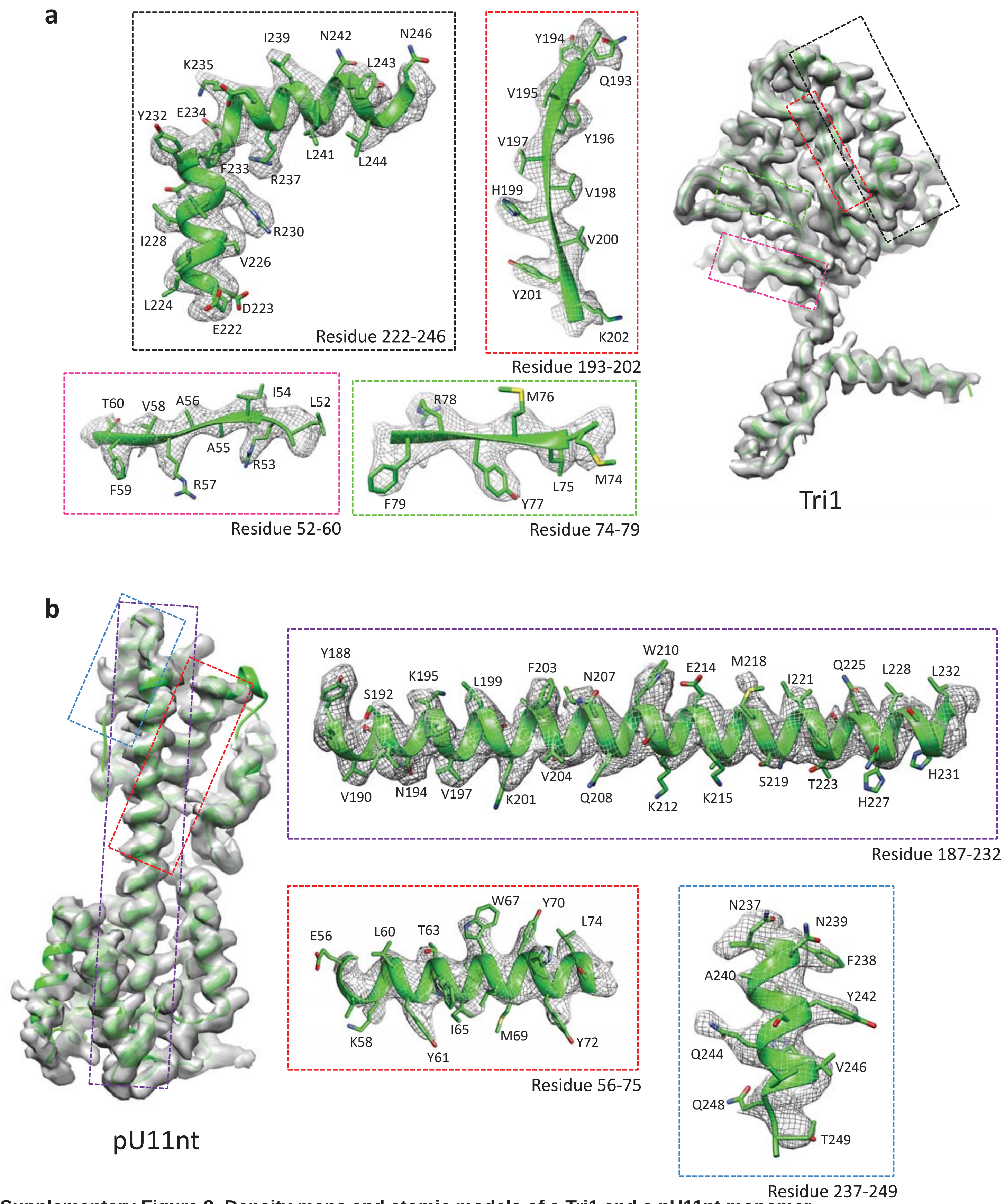

Supplementary Figure 8. Density maps and atomic models of a Tri1 and a pU11nt monomer.
(**a-b**) The density maps (gray) of a Tri1 (a) and a pU11nt (*e.g.*, pU11-a-2) (b) (segmented out from triplex Tb of the 3-fold axis sub-particle reconstruction) at 3.77 Å are superposed with their atomic models (ribbon). Boxed regions are enlarged, with density shown as gray mesh and atomic models as ribbon/sticks, in boxes with corresponding color edges.

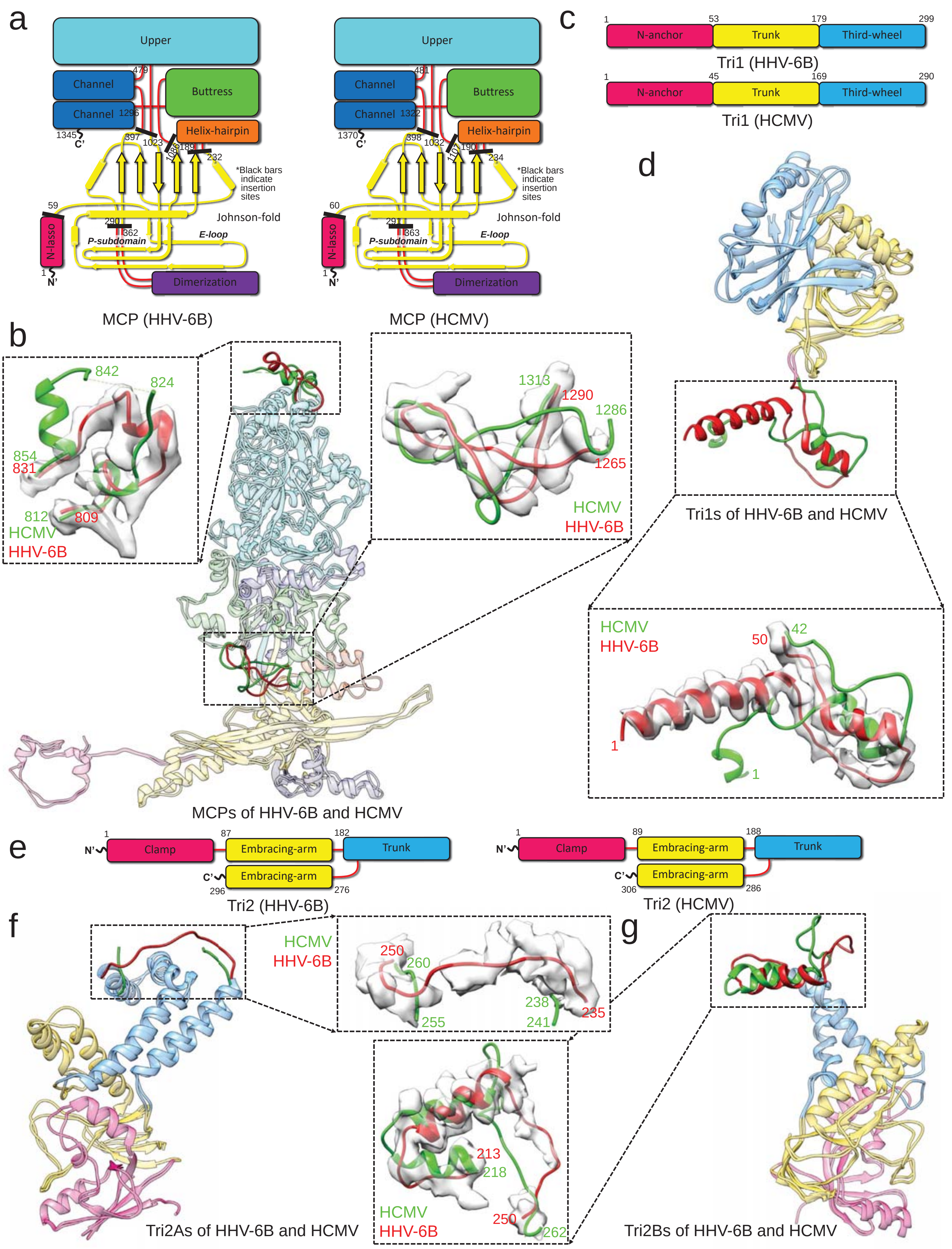
a
Upper
Channel
Buttress
Channel
Helix-hairpin
*Black bars indicate insertion sites
Johnson-fold
N-lasso
P-subdomain
E-loop
Dimerization
MCP (HHV-6B)
MCP (HCMV)
c
N-anchor
Trunk
Third-wheel
Tri1 (HHV-6B)
Tri1 (HCMV)
b
HCMV
HHV-6B
MCPs of HHV-6B and HCMV
d
Tri1s of HHV-6B and HCMV
e
Clamp
Embracing-arm
Trunk
Tri2 (HHV-6B)
Tri2 (HCMV)
f
g
Tri2As of HHV-6B and HCMV
Tri2Bs of HHV-6B and HCMV

Supplementary Figure 9. Structural differences between corresponding HHV-6B and HCMV subunits of MCP, Tri1, Tri2A, and Tri2B.
(**a**) Schematics of MCP domain organization in HHV-6B and HCMV. (**b**) Superposition of the structures of the C6 subunit of MCP from HHV-6B and HCMV colored as in (a) by domains. Structural differences are located mainly in their upper (left inset) and buttress (right inset) domains, which are shown together with the HHV-6B cryoEM map (semi-transparent gray) in the insets. (**c**) Schematics of Tri1 domain organization in HHV-6B and HCMV. (**d**) Superposition of the structures of Tri1 subunit of triplex Td from HHV-6B and HCMV. Structural differences are located in their N-anchor domains, which is shown together with the HHV-6B cryoEM map (semi-transparent gray) in the inset. (**e**) Schematics of Tri2 domain organization in HHV-6B and HCMV. (**f, g**) Superposition of structures of the Tri2A (f) and Tri2B (g) subunits from triplex Td from HHV-6B and HCMV. Structural differences are located mainly in their embracing-arm domains, which are shown together with the HHV-6B cryoEM map (semi-transparent gray).

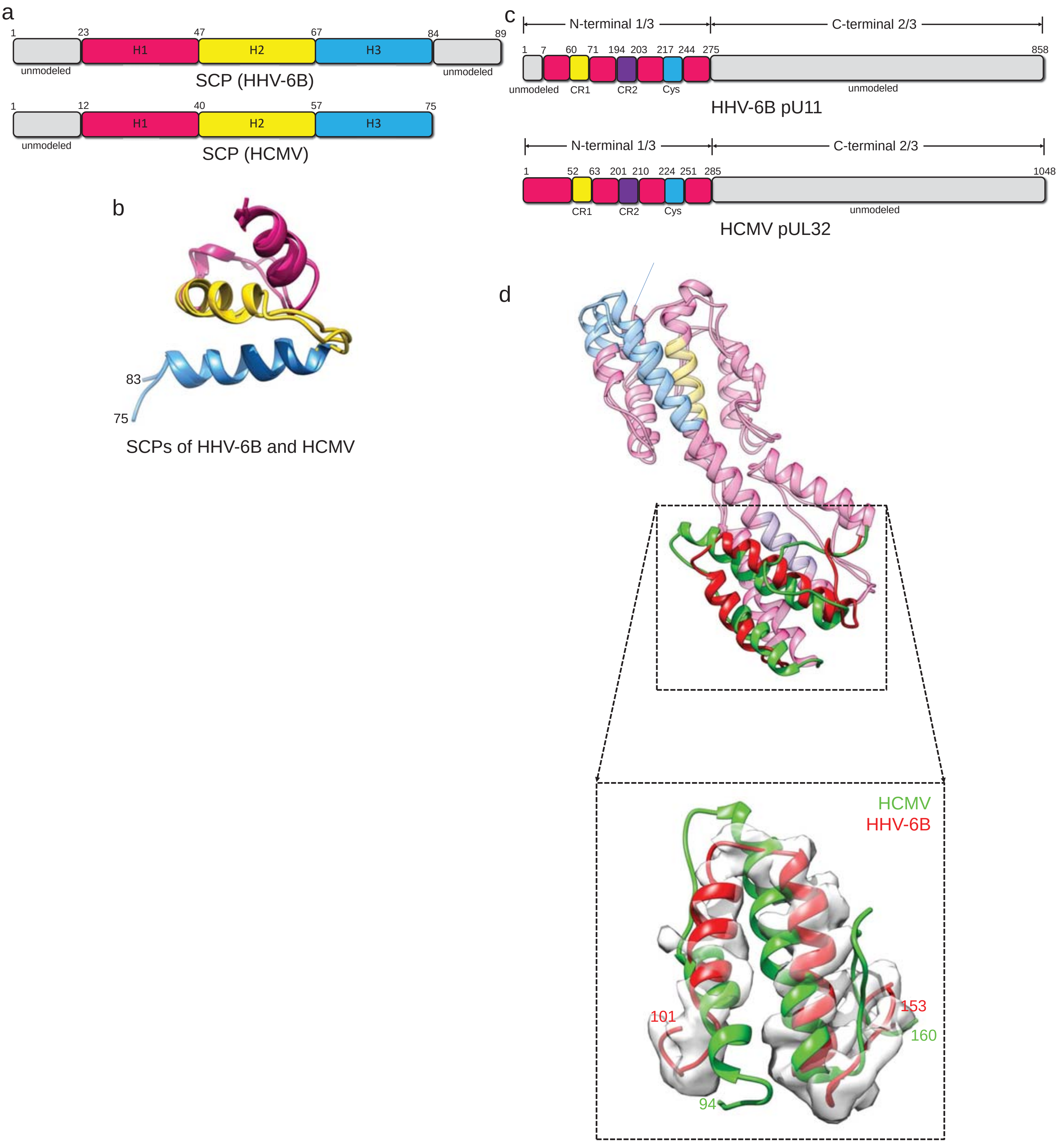

Supplementary Figure 10. Structural differences between corresponding HHV-6B and HCMV SCP subunits, and between HHV-6B pU11nt and HCMV pUL32nt.

(**a**) Schematics of SCP domain organization in HHV-6B and HCMV. (**b**) Superposition of structures of the C6 conformer of SCP from HHV-6B and HCMV showing minor differences between the two structures. (**c**) Schematics of domain organization in HHV-6B pU11 and HCMV pUL32. (**d**) Superposition of the structures of HHV-6B pU11nt (the a-2 conformer bound to triplex Tb) and HCMV pUL32nt (the c conformer bound to triplex Tb). Structural differences are located mainly in the lower domain, which is shown together with the HHV-6B cryoEM map (semi-transparent gray).

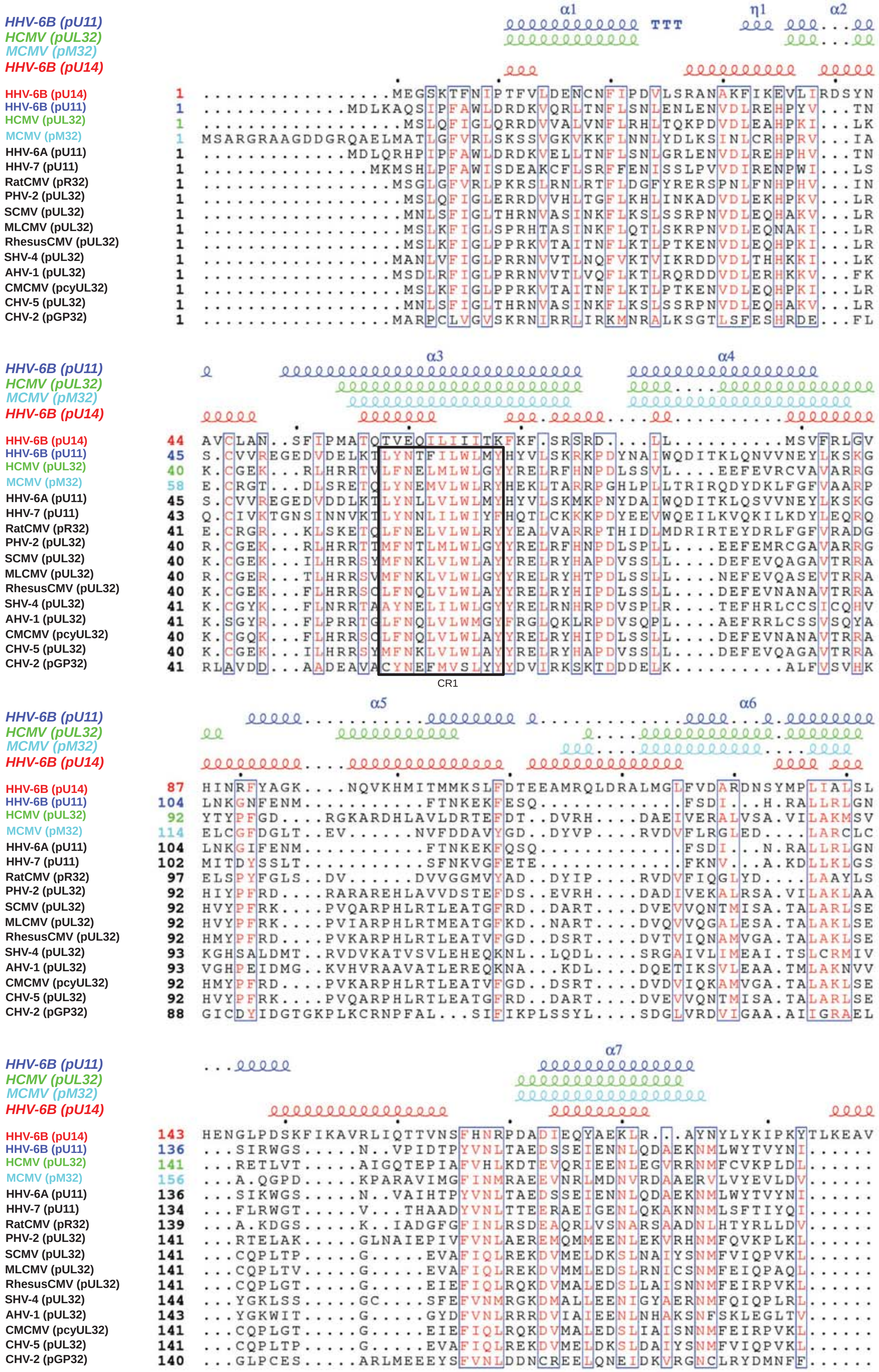

HHV-6B (pU11)
HCMV (pUL32)
MCMV (pM32)
HHV-6B (pU14)
α1
TTT
η1
α2
α3
α4
α5
α6
α7
HHV-6B (pU14)
HHV-6B (pU11)
HCMV (pUL32)
MCMV (pM32)
HHV-6A (pU11)
HHV-7 (pU11)
RatCMV (pR32)
PHV-2 (pUL32)
SCMV (pUL32)
MLCMV (pUL32)
RhesusCMV (pUL32)
SHV-4 (pUL32)
AHV-1 (pUL32)
CMCMV (pcyUL32)
CHV-5 (pUL32)
CHV-2 (pGP32)
CR1

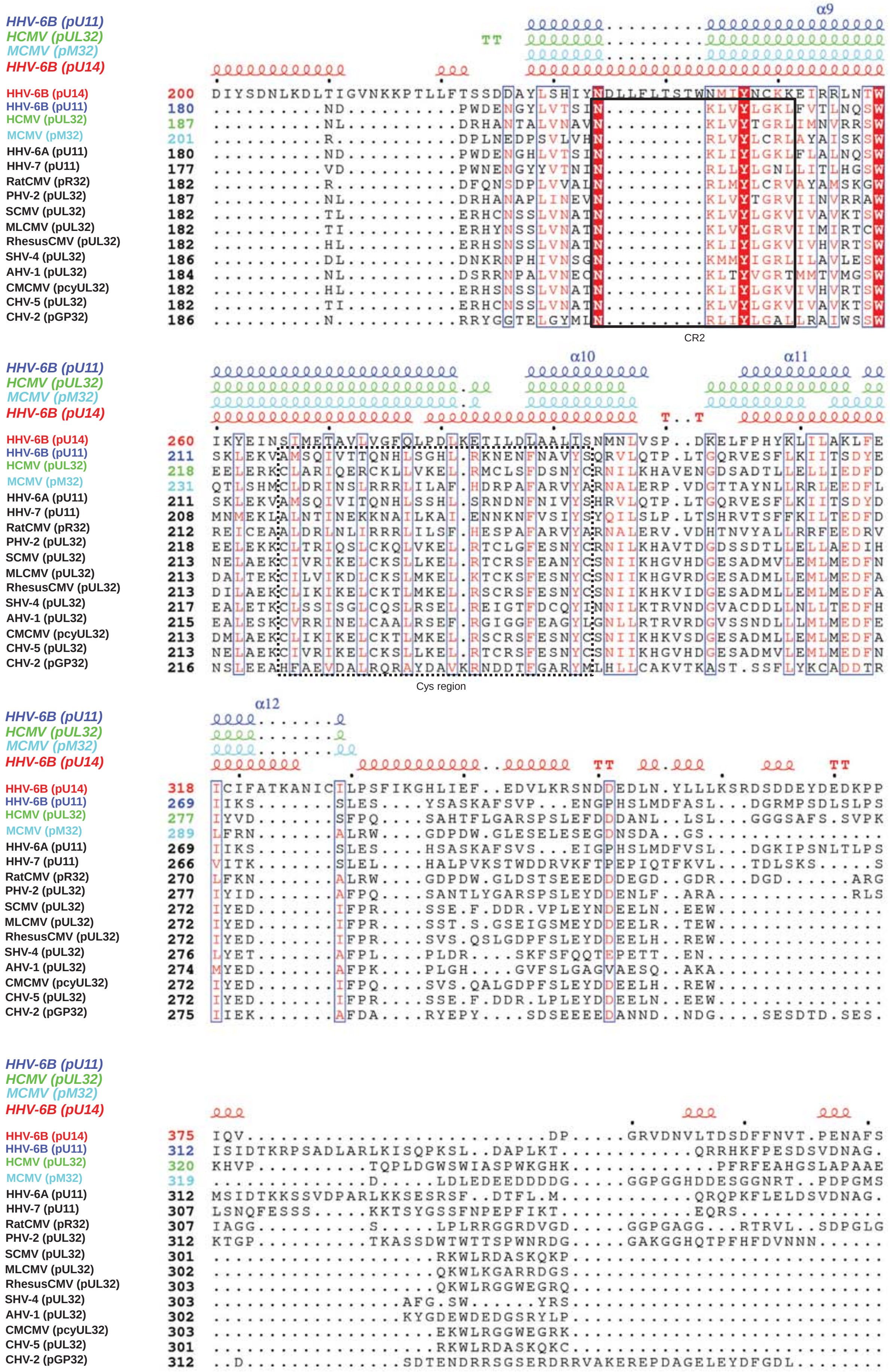

HHV-6B (pU11)
HCMV (pUL32)
MCMV (pM32)
HHV-6B (pU14)
α9
α10
α11
α12
CR2
Cys region
HHV-6B (pU14)
HHV-6B (pU11)
HCMV (pUL32)
MCMV (pM32)
HHV-6A (pU11)
HHV-7 (pU11)
RatCMV (pR32)
PHV-2 (pUL32)
SCMV (pUL32)
MLCMV (pUL32)
RhesusCMV (pUL32)
SHV-4 (pUL32)
AHV-1 (pUL32)
CMCMV (pcyUL32)
CHV-5 (pUL32)
CHV-2 (pGP32)

HHV-6B (pU11)
HCMV (pUL32)
MCMV (pM32)
HHV-6B (pU14)

```
HHV-6B (pU14)      402  SIAI.....................MPISYDK........................TIDV
HHV-6B (pU11)      357  ..G.KILIKKETLGGRDVRATTPV.SSVSLMSGVEP.............LSSLTSTNLDL
HCMV (pUL32)       357  AHAARS...................AAVGYYDEEE.........KRRERQKRVDDEVVQR
MCMV (pM32)        353  LHDDTGIANTCLIGGDDEDCGGGSGRCLEFDPDSERCLGVKMVNGRALRWWNPTGIMV..
HHV-6A (pU11)      356  ..G.KILIKEATLGGEDVKATTPA.SSVSLMSGVESPSSFTSTNL.DLPLSSFTSTNLDL
HHV-7 (pU11)       337  ..N.NTLSKDLFVGSEDG...........LLSSVKKD....SMIL.DEPRNS...TSINN
RatCMV (pR32)      342  RGDDRGKETPGRS......ASRG............RGGSVREDDGGRSSWRSATDGEL..
PHV-2 (pUL32)      351  ............................PFLD...................KKTREEIER
SCMV (pUL32)       313  ............................DYESKFE.........S...............
MLCMV (pUL32)      314  ............................PEYNKWD.........T...............
RhesusCMV (pUL32)  315  ............................TKDDDDD.........N...............
SHV-4 (pUL32)           ............................................................
AHV-1 (pUL32)           ............................................................
CMCMV (pcyUL32)    315  ............................TKDDDDD.........D...............
CHV-5 (pUL32)      313  ............................DYDSKLE.........T...............
CHV-2 (pGP32)      348  .................................FG.SDGLNDVNN...............
```

HHV-6B (pU11)
HCMV (pUL32)
MCMV (pM32)
HHV-6B (pU14)

```
                        TT                  
HHV-6B (pU14)      417  EDNEI.QVLEVEMQSLSAVVY.......GAVASKYGLSLEQVIRKLNQNEGR........
HHV-6B (pU11)      400  RDKSHGNYRIGPSGILDFGVK.........LPAEA...........QSNTGD......VD
HCMV (pUL32)       389  EKQQL.KAWEERQQNLQQRQQQP......PPPARKPS.ASRRLFGSSADEDDDDDDDEKN
MCMV (pM32)        411  .DNEA.AVWIDEHG...RVMDKP........PP.......KELRKASSDDGGN.....KK
HHV-6A (pU11)      411  RDKSHGNYKIGPSGILDFNVK.........FPPDA...........QLNTND......VD
HHV-7 (pU11)       375  SKK...MHRILQTEILDLTDQ.........TM.................HRP......ED
RatCMV (pR32)      382  .SDGE.RSWSGDGDGFDSLLDVP.................RSLRAEPDAAGDG.....DR
PHV-2 (pUL32)      364  EKKRD.REWERKQKALRDAATQGRTADAGRTPMRTRR.GSPSLFRESSDDDD.DDDIDGR
SCMV (pUL32)       321  ...KK.Y.....................................................
MLCMV (pUL32)      322  ...KK.DKWDTKKDEWDTKKD............................ES.........
RhesusCMV (pUL32)  323  ............GQLLTTK...............................K.........
SHV-4 (pUL32)      311  ..................SL............................PES.........
AHV-1 (pUL32)      317  ..................VFPK........................PPDSG.........
CMCMV (pcyUL32)    323  ............NQLFTTK...............................K.........
CHV-5 (pUL32)      321  ...KK.Y.....................................................
CHV-2 (pGP32)      359  ..............LLKNITI.........TPKRILSDTAYRLRGLADDHDK...RHEER
```

HHV-6B (pU11)
HCMV (pUL32)
MCMV (pM32)
HHV-6B (pU14)

```
HHV-6B (pU14)      461  T.....SSRASPSHSTST.....................VPYSPPQRHRSTPTSILRQRV
HHV-6B (pU11)      434  LLQDKTSIRSPSSGITDVVNG...LAN.................................
HCMV (pUL32)       441  IFTPIKKPGTSGKGAASGGGVSSIFSGLLSSGSQKPTSGPLNIPQQQQRHAAFSLVSPQV
MCMV (pM32)        446  NPPPKKNVTPPVSGSNSVGGGVQTPS.................................T
HHV-6A (pU11)      445  LLQDKTSIGSPSSGITDVVNG...FAN.................................
HHV-7 (pU11)       400  KVNQFNEIAVAPDGINQVIDT...LSK.................................
RatCMV (pR32)      418  KAGAKRR........ASVGAA.......................................
PHV-2 (pUL32)      421  TFAPIRNPSTRPSAPGSA..GSGIFSAPK........LGSNI..APPYRQTAFDILSPIP
SCMV (pUL32)       324  ......................................DFKKPSGKTDRQVAFSMLSPTE
MLCMV (pUL32)      341  ......................................EKKHSDTKTDRQMAFSMLSPTD
RhesusCMV (pUL32)  331  ......................................QERKVPDKSERQYAFSLLSPTK
SHV-4 (pUL32)      316  LLADLKDPAVR..........................YKVFDIREPTERHKAFEILSDDD
AHV-1 (pUL32)      326  DLLDLKDPAVR..........................RHVFDIEP...........LDDE
CMCMV (pcyUL32)    331  ......................................QERKVPDKSERQYAFSLLSPTK
CHV-5 (pUL32)      324  ......................................DFKKPSGKTDRQVAFSMLSPTE
CHV-2 (pGP32)      393  P..DAGRADAKPSGK..DG.ETSR............................YTLAD...
```

HHV-6B (pU11)
HCMV (pUL32)
MCMV (pM32)
HHV-6B (pU14)

```
HHV-6B (pU14)      495  ....PIRSNSRSS............................SVSFSQEDSNRSH...YS.
HHV-6B (pU11)      458  ...LNLRQNKSDVSRPWSKNTAANADVFDPVHRLVSEQTGTPFVLNNSDVAGSEAKLTTH
HCMV (pUL32)       501  TKASPGR...VRRD..........SAWDV....RPLTETRGDLFSGDEDSDS.......S
MCMV (pM32)        473  ..ASGKR...T......................GKKKEGGGGYLLRSRSTDDDEVRKMKK
HHV-6A (pU11)      469  ...LNLHQNKSNVSPPWSRNTAANADFLDPVHRFVPEQTGTPFVLNNSDVAGSEEKHTTY
HHV-7 (pU11)       424  ...LDLHNSNKVIDIVSSPKV..NV.VQLPKNKI...DYHSTFFLPENEVNRQNGVQS.R
RatCMV (pR32)      431  ..........S......................AADRPRARTYWLESGGDPSSGGAPGPE
PHV-2 (pUL32)      469  TRASDNR...LRRD..........AAWES...VTPLTVTRSSDWLTEEDSGSDD...YQL
SCMV (pUL32)       346  ..KRDFWEKPVTT....................KPSAKRRVTFWSDDENDWK.E...KK.
MLCMV (pUL32)      363  T.KADFWEKPTSATVSWEKPALTTTAWKKSAATTVPSAKRVTFWPDDDKEWE.N...TE.
RhesusCMV (pUL32)  353  Q.SSQFWDTKP....................KTPHPVSKRVTFWSDDENQGRAK...DE.
SHV-4 (pUL32)      350  EK.PDYSQKKRDRD...........................SWWEVDSTDDSTK...KAS
AHV-1 (pUL32)      349  EG.FGYSQNRRHRD...........................YTWDVQYGGDQDK...PKR
CMCMV (pcyUL32)    353  LTSSQLWDTKP....................KTPHPVSKRVTFWSDDENQGRTK...GE.
CHV-5 (pUL32)      346  ..KRDFWEKPVAT....................KPSAKRRVTFWSDDESDWK.E...NK.
CHV-2 (pGP32)      417  ......................TSDLYKIANVPIPPSPEEGGEFLNEPFEGRPDRR.TQL
```

HHV-6B (pU11)
HCMV (pUL32)
MCMV (pM32)
HHV-6B (pU14)

```
HHV-6B (pU14)       519  ..............DETNISDYSYPMADLEL....................EDEEPMEDH
HHV-6B (pU11)       515  ..STETG...VSPHNVSLIKDLRDKDGFRK.....QKKLDLLGS......WTKEKNDKAI
HCMV (pUL32)        537  DGYPPNR...QDPRFTDTLVDITDTETSA.....KPPVTTAYKFEQPTLTFGAGVNV...
MCMV (pM32)         506  DGTIDDR.......ADR...ELKMALQKAR.ESTADSDLSTILPRTEPLRKVAFVGDPVA
HHV-6A (pU11)       526  ..STETG...VSPRNVFLIKDLRGKDGFRK.....QKQSDIPKS......LTKERNDKAI
HHV-7 (pU11)        474  ..DQLSK...NSTNDLQKILELRERIKTIK.....QNNEDIFK.......LPSEKRRKEI
RatCMV (pR32)       459  RVFVSDG.......KTV...TLPVALDAAS.PSPASS...T...RPPPERPGTEIGTDDV
PHV-2 (pUL32)       510  RGYAPTGPRPSSSRVSNTLVDLTTPGGNA.....AQPQQTSYKFETVPLVTPQPSSI...
SCMV (pUL32)        379  ..........AEKTKGDSMLDLGDSLESLKLDSTMDRPTSTYRFEPPKLVQSSEYN....
MLCMV (pUL32)       417  ..........TDNVKVDKLVDLGNGLEGLKLDSKQ.HTIPVYKFEPPKLAPSTEHN....
RhesusCMV (pUL32)   388  ..........TDDVKVDTLVDFDGGLGSLKLDDDDKPSTSTYKFELPKFIQTPILSS...
SHV-4 (pUL32)       379  .....AL.......DSSGLLDLEKQLEKLDVG...GVPTQAFVFEPPFKPTETVSQ....
AHV-1 (pUL32)       378  RGSSPKG.......ASGSVEDLVDRLGDLDVK...GGKKPMFVFEPPSIPMPSTVT....
CMCMV (pcyUL32)     389  ..........TDDVKVDTLVDFDGGLGSLKLDDDDKPSTSTYKFELPKFIQTPLLNS...
CHV-5 (pUL32)       379  ..........AEKTKGDSMLDLGDSLESLKLDSTMDQPMSTYKFEPPKLVQSSEYN....
CHV-2 (pGP32)       454  DNDI..........DQMNIRPLSSAVQPKIVTFAHPEPSKAVTFSPPKINLPLTAS....
```

HHV-6B (pU11)
HCMV (pUL32)
MCMV (pM32)
HHV-6B (pU14)

```
HHV-6B (pU14)       545  PHS.......PQSTSSNN..SMSR.....QSRALQNGQRRRAPTMVPSSQTR.RQNN...
HHV-6B (pU11)       559  VHS.......REVTGDSGDATET.....................VTARDSPVLRKT....
HCMV (pUL32)        586  PAG......AGAAILTPTPVNPS.................TA..PAPAPTPTFAGTQTPV
MCMV (pM32)         555  AFGDTVRTTSSSKGFDDGPFTTQGASVLLPPPLGGPGSTLT....LPPDLPDLSGVLADE
HHV-6A (pU11)       570  MHS.......REVTGDSGDATET.....................VGARNSPALRKI....
HHV-7 (pU11)        517  VHE.......NLQSFDDEHNEMS.....................LPPQDQKSIKQK....
RatCMV (pR32)       502  VFP.......PISYLDERYFRPS......GPRVDGPGGREER..DLSPLRPR........
PHV-2 (pUL32)       562  L........MPIRLGTQTPSNPS.................AA..PAPASTPTLGGNSESP
SCMV (pUL32)        425  PFL......QPLPNFTNE..YES.................FR..PLPPEE..........
MLCMV (pUL32)       462  PFI......QPIPHLEP...TSS.................FQ..PIKIDN..........
RhesusCMV (pUL32)   435  PFA......TQFSN..............................P...............
SHV-4 (pUL32)       420  PFA......PVSEQGDLK...TS.................LT..PET.............
AHV-1 (pUL32)       424  PFS......QALGSPSSD...VT.................VL..H...............
CMCMV (pcyUL32)     436  PFA......TQFSN..............................P...............
CHV-5 (pUL32)       425  PFL......QPLPKFTNE..YES.................FK..PLPPDD..........
CHV-2 (pGP32)       500  PFSVLRKTDAPAGQGDVR....................RVET..PEPSSADDFSLISESE
```

HHV-6B (pU11)
HCMV (pUL32)
MCMV (pM32)
HHV-6B (pU14)

```
HHV-6B (pU14)       587  ..................AR......PR................................
HHV-6B (pU11)       587  ................KHAN................DIFAGLNKKYARDVSRGGKGNSRD
HCMV (pUL32)        621  ..NGNSPWAPTAPLPGD.MNPANWPRERAWALKNPHLAYNPFRMPTT...........ST
MCMV (pM32)         611  ..DVQD............................................SRYGKI....
HHV-6A (pU11)       598  ................KQAN................DFFAGLNKKNDRDVLRGGKGNRKD
HHV-7 (pU11)        545  ..............NGNKAN................SSTKTLNMIGTNDVNA........
RatCMV (pR32)       539  ..................................................SLTGKA....
PHV-2 (pUL32)       595  ..LISIDSGTTPPMTEQQAAAIRNNPNRAWAVKVPWLAVNDFAPYAQ...........ST
SCMV (pUL32)        448  .................KPTVADWTNTFYPGLSTP...LVPLSDEGKTDVSDEGRTDGSD
MLCMV (pUL32)       484  ..................RGLFDWSNELSSLKPTDAPTIVPVFP..............PD
RhesusCMV (pUL32)   444  ...................LFATGSSETIPGLETPLKEV...........SG........
SHV-4 (pUL32)       439  ...............EEVASVE......................................
AHV-1 (pUL32)       441  ...............DEQAKRS......................................
CMCMV (pcyUL32)     445  ...................LFATGSSETIPGLETPLKEV...........SD........
CHV-5 (pUL32)       448  .................KTTVADWTKTFYSGLPTS...IVPLSDEGKTDVSDEGKTDGSD
CHV-2 (pGP32)       538  APDAESDVPAEAPDSS..K..................AIDD..........IVGKLQQM.
```

HHV-6B (pU11)
HCMV (pUL32)
MCMV (pM32)
HHV-6B (pU14)

```
HHV-6B (pU14)       591  ..........RVA.................RRL....TEMMNDAR...L...........
HHV-6B (pU11)       615  LYSGGNAEKKETSGKFNVDKEMTQNEQEPLPNL..MEAARN..AGEEQYVQAGLGQRVNK
HCMV (pUL32)        667  AS......QNTVSTT....PRRPS......TPRAAVTQTASRDAA..DEVWALRDQTAES
MCMV (pM32)         621  ..........PKSR.TKKHPTFPEN...YKQRP...P..PHNKDD..EYYWDETGDNMPV
HHV-6A (pU11)       626  LHSGGNAKKKEMSGKFNVDKEMTRNGQEPSRSL..MGDSRN..AGDGQYIQAGLGQRVNN
HHV-7 (pU11)        567  .....SMKEKESASSAKKNQLVKDVKWTPSSSL..LDLSRRNDLLQKELFESGLGEKVKK
RatCMV (pR32)       545  ..........VRPRKAAAQRNSGAS...YAKRR...PTVRSDDED..EYYWDDLGEADDG
PHV-2 (pUL32)       642  KP...TLTPAALSTQ....PLVPTSVWQPSPNKSSIGSTRLSSAT..RT....PASIINT
SCMV (pUL32)        488  SS...DVEK.TVSDFSKIDFTDPNSNFSPAKRREGNSGTEGSD.............SDGE
MLCMV (pUL32)       512  SD...VETKGAASDLSDVDFTDPNSNFSPAKRRDPSETEASDDD..........DDGVRS
RhesusCMV (pUL32)   466  LD...SISDYETKTPPASDYSDRESSFSPNKRREVKSEAEISE.............SEYE
SHV-4 (pUL32)       446  ................................KEKKENEEGEDED..DEESIET......
AHV-1 (pUL32)       448  ................................AGSGDGDQGDDED..DEVAEEEV.EEEE
CMCMV (pcyUL32)     467  LD...NMSDRERKTPPGSDYSDRESSFSPNKRREVKSEAEMSE.............SEYE
CHV-5 (pUL32)       488  SS...DVEKKPVFDLSDIDFTDPNSSFSPAKRREVNSGTEGSD.............SDGE
CHV-2 (pGP32)
                         ............................................................
```

HHV-6B (pU11)
HCMV (pUL32)
MCMV (pM32)
HHV-6B (pU14)

```
HHV-6B (pU14)             ............................................................
HHV-6B (pU11)        671  I.............LAEFTNLISLGEK.......GIQDILHNQSGTELKLPTENKLGRES
HCMV (pUL32)         709  PVEDSEEEDDD...SSDTGSVVSLGHTTPSSDY.NNDVISPPSQTPEQSTPS........
MCMV (pM32)          660  GEDG..........GGVLEDLR........KGLEGIDL..KTGGG...............
HHV-6A (pU11)        682  L.............LSQFTNLISLGEK.......GIEDILQNQRGTELKLATENKSGRES
HHV-7 (pU11)         620  L.............LTDFTDTISLEER.......SLKDVLEPPKKTDVSNIA......TF
RatCMV (pR32)        587  .VAE..........DDDFERIQ........RQLQGLNINSPTAGG...............
PHV-2 (pUL32)        689  DSEDSDSEEAT...SSDASSVKSQRTVPPKRPF.TFK.......TPTMTGP.........
SCMV (pUL32)         531  SVTDLFSD.DG...VSDAESVVSVPPETQ....................P.K........
MLCMV (pUL32)        559  EVADLFSDD.....GSDAESVASVASVAV....................TPK........
RhesusCMV (pUL32)    510  DIASGDGDEEA....SDAESVVSVASIPPATPV............PPVSDPK........
SHV-4 (pUL32)        466  ............................IDVADKVKQIV............D........
AHV-1 (pUL32)        473  EPDEGEDEEDGAGGGGDDGAPAPAAPVTPEEAAANIQKIV............E........
CMCMV (pcyUL32)      511  DIASGDGDEEG...VSDAESVVSVASMPPATPV............PPVSDPK........
CHV-5 (pUL32)        532  SVTDLFSDNDG...VSDAESVVSVRPEKP....................PPK........
CHV-2 (pGP32)             ............................................................
```

HHV-6B (pU11)
HCMV (pUL32)
MCMV (pM32)
HHV-6B (pU14)

```
HHV-6B (pU14)             ............................................................
HHV-6B (pU11)        711  EEANVERILEVSDPQNLFKNFKLQNDLDSVQSPFRLPNADLSRDLDSVSFKDALDVKLPG
HCMV (pUL32)         757  ..RIRKAKLSSPMTTT...........STSQKPVLGKR...VATPH.ASARAQTVTSTPV
MCMV (pM32)          685  ......GSLQPPLSQQ............FAGSPFAGSDGDGGGLVK.KSSSS........
HHV-6A (pU11)        722  EEANVQKILEFSNPQDMFKNFRLQNNLDSVQSPFRLPDVDLSRELDSASFKDALDLKLPG
HHV-7 (pU11)         654  NDNNLKNLLNSRKRDPLFQNFSFTEKMQPVRSPFFLPNAEIQD.FDSGSLLTGKET....
RatCMV (pR32)        613  ......PQFAPPLSRG............MPSPPSFLPAGDAVGGPD.ASDSSG.......
PHV-2 (pUL32)        729  ..QKSTRTFTPPVIPS...........TGTQTPVFGAR...VRTPA.PTARAQTVSSSPV
SCMV (pUL32)         558  ..AKRKILVAK.......SPVKPKTKPIPKPKPSLGPF...PPLTP....SPNIVEIKAP
MLCMV (pUL32)        586  ..AKRKLVLEPPK..........TVKLQPKPKPSLGPF...PPVDP....LAAIDTRRIL
RhesusCMV (pUL32)    546  ..AKRRLILKPK...........TK.IIPKPKPSFGPF...TSVTS....PSTSV.....
SHV-4 (pUL32)        478  ..AIKDNEES..................................................
AHV-1 (pUL32)        514  ..AIKEDQLSP.................................................
CMCMV (pcyUL32)      548  ..AKRRLILKPK...........TKKNVPKPKPSLGPF...TSVTS....PSTSV.....
CHV-5 (pUL32)        561  ..TDRQILVAKKPTVRPKSRVTPKPKVIPKPKPSLGPF...PQAS......PNIVEIKPP
CHV-2 (pGP32)             ............................................................
```

HHV-6B (pU11)
HCMV (pUL32)
MCMV (pM32)
HHV-6B (pU14)

```
HHV-6B (pU14)             ............................................................
HHV-6B (pU11)        771  NGEREIDLAL.QKVKAGERETSDFKVGQDETLIPTQLMKVETPEE...............
HCMV (pUL32)         800  QGRLEKQVS...G...............TPSTVPATLLQPQPASSKTTSSRNVTSGAGTS
MCMV (pM32)               ............................................................
HHV-6A (pU11)        782  NGEREIDLAL.EKVKVGETETSDLKVGQDESFVPAQLMKVETPEE...............
HHV-7 (pU11)         709  ...QNTIFGASKAQEDGDKD................LIDLENSVQK..............
RatCMV (pR32)        647  ..................................VTVLSND.....TGDRRRHKSGSGGR
PHV-2 (pUL32)        772  SPAPPVKSQ...AQPMGSRI......S.TAPTMPASFLTPANVPKT.PS...........
SCMV (pUL32)         602  D......D....IKPLESKV......P.KTVKAPPTVVKPATLN................
MLCMV (pUL32)        627  K......PT...VKPVTPKP.........PVATKPVVLKPATLQ................
RhesusCMV (pUL32)    580  ............LKPRKVIK......A.TPLTVDGSVLKPASFS................
SHV-4 (pUL32)             ............................................................
AHV-1 (pUL32)             ............................................................
CMCMV (pcyUL32)      583  ............LKPRKVIK......A.TPITLDGSVIKPANFS................
CHV-5 (pUL32)        610  E...........IKSPEIKA......P.KSAKATP.VVKPATLV................
CHV-2 (pGP32)             ............................................................
```

HHV-6B (pU11)
HCMV (pUL32)
MCMV (pM32)
HHV-6B (pU14)

```
HHV-6B (pU14)             ............................................................
HHV-6B (pU11)        815  ..................KDDVIEKMV.................................
HCMV (pUL32)         842  SASSARQPSASASVLSPTEDDVVSPATSPLSMLSSASPSPAKSAPPSPVKGRGSRVGVPS
MCMV (pM32)               ............................................................
HHV-6A (pU11)        826  ..................KDDVIERMV.................................
HHV-7 (pU11)         736  ..................DDDIVNKLV.................................
RatCMV (pR32)             ............................................................
PHV-2 (pUL32)        810  ...PLAVPRPATTAVKTPASATSSPLRSPASMLSSAPTSAPSSATSSPVKL..TIRPQPS
SCMV (pUL32)         629  ......VPTAIA.........TQAKAV....KPSSDAGTAAKVAK...............
MLCMV (pUL32)        653  ......VPATTT.........ASSAKT....ALN..TVSKALGST...............
RhesusCMV (pUL32)    605  ......APIPGK.........DV.....................................
SHV-4 (pUL32)             ............................................................
AHV-1 (pUL32)             ............................................................
CMCMV (pcyUL32)      608  ......APGSGK.........DV.....................................
CHV-5 (pUL32)        635  ......APTATE.........T......................................
CHV-2 (pGP32)             ............................................................
```

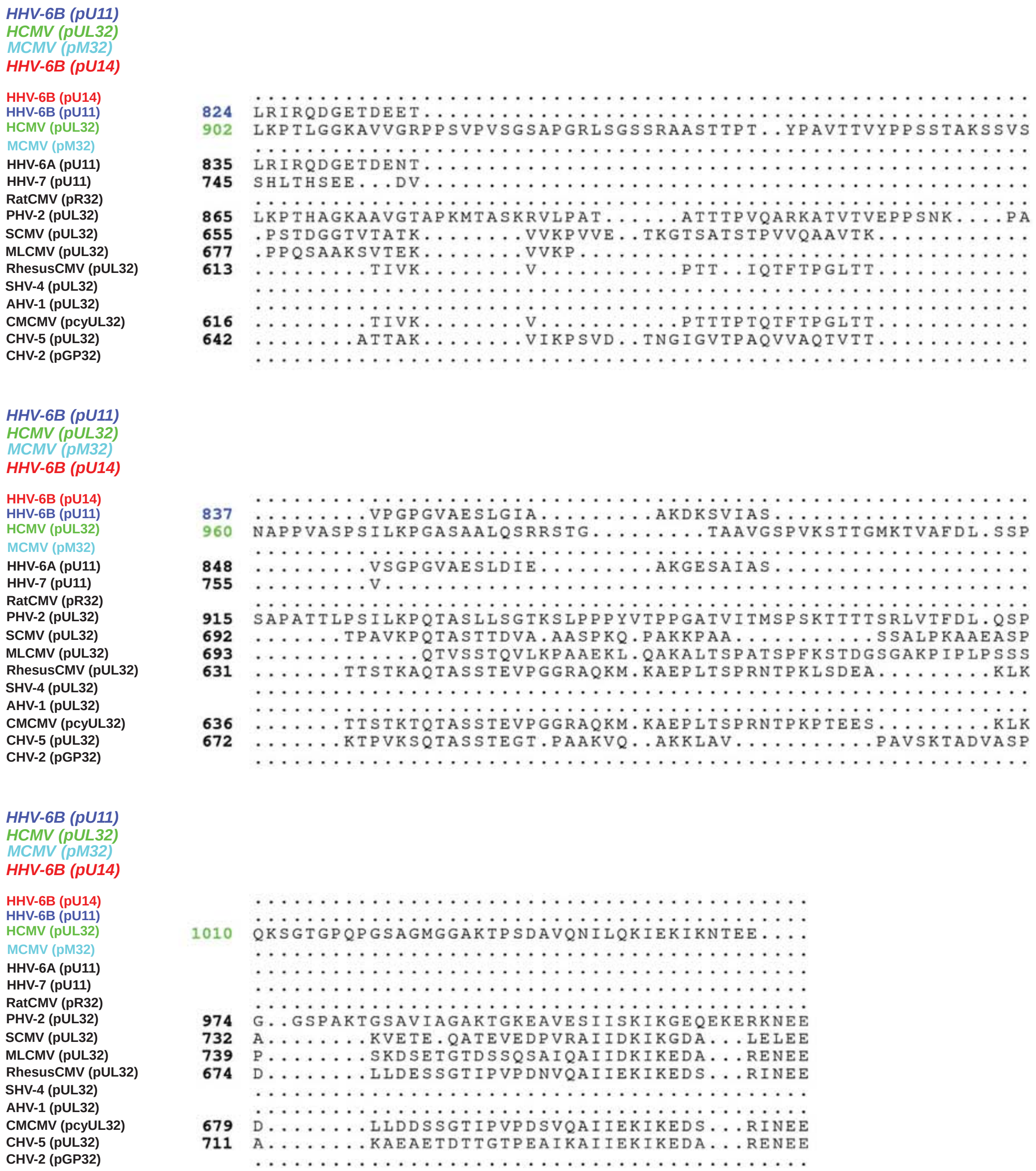

Supplementary Figure 11. Secondary structure for pU11nt, pUL32nt, pM32nt, and pU14nt, and pp150 sequence alignment for 15 CMVs.

Schematic representations of the secondary structure alignment for pU11nt, pUL32nt, pM32nt, and pU14nt[2], and amino acid sequences of pp150s from HHV-6B[3], HHV-6A[3], HHV-7[4], Human cytomegalovirus (HCMV)[5], Murine cytomegalovirus (MCMV)[6], Rat cytomegalovirus Maastricht (RatCMV)[7], Chimpanzee cytomegalovirus (PHV-2)[8], Simian cytomegalovirus (SCMV)[9], Mandrillus leucophaeus cytomegalovirus (MLCMV)[10], Rhesus cytomegalovirus (RhesusCMV)[11], Saimiriine β-herpesvirus 4 (SHV-4)[12], Aotine β-herpesvirus 1 (AHV-1)[12], Cynomolgus macaque cytomegalovirus (CMCMV)[13], Cercopithecine β-herpesvirus 5 (CHV-5)[12], Caviid β-herpesvirus 2 (CHV-2)[14] analyzed and displayed by *ESPript* 3.0[15]. Spiral represents α-helix.

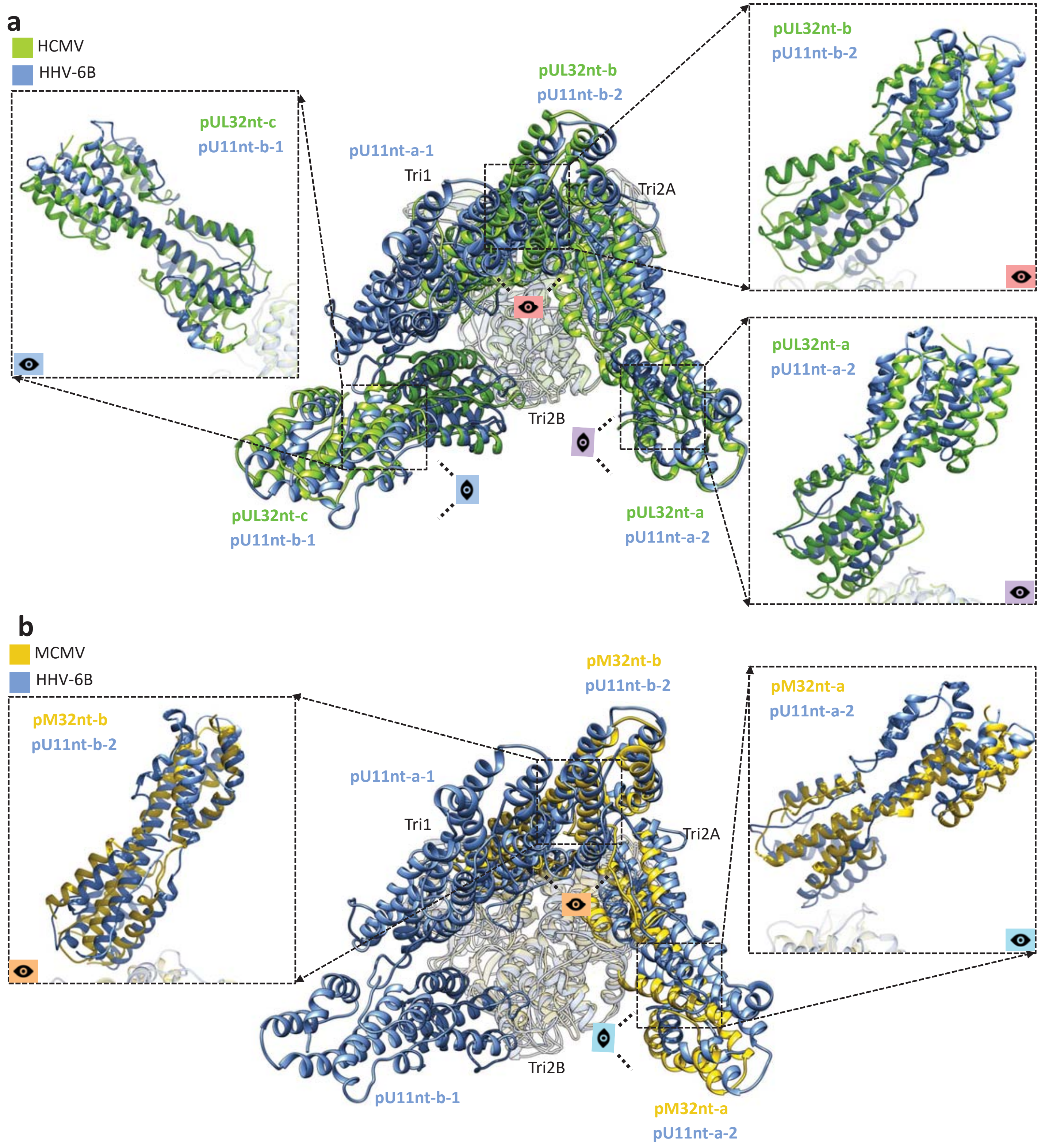

Supplementary Figure 12. Structural differences of CATCs in HHV-6B, HCMV, and MCMV.
(**a**) The atomic models of triplex Te (gray) and pU11 in HHV-6B (light blue) and corresponding structures in HCMV (green) are superposed together as rigid bodies to show differences in their Te-associated CATCs. Conformers b-2 of CATC (*i.e.*, pU11nt-b-2 of HHV-6B and pUL32nt-b in HCMV) exhibit major translational and rotational displacements (upper right panel), while conformers a-2 (pU11nt-a-2 and pUL32nt-a, bottom right panel) and b-1 (pU11nt-b-1 and pUL32nt-c, left panel) exhibit relatively minor rotational displacements between HHV-6B and HCMV. Conformer a-1 (pU11nt-a-1) exists only in HHV-6B. (**b**) The atomic models of triplex Te (gray) and pU11 in HHV-6B (blue) and the corresponding structures in MCMV (gold) are aligned together as rigid bodies to show differences in their associated CATCs. The a-2 CATC conformers (pU11nt-a-2 in HHV-6B and pM32nt-a in MCMV, right panel) exhibit major translational and rotational displacements, while conformers b-2 (pU11nt-b-2 and pM32nt-b, left panel) exhibit relatively minor rotational displacements between HHV-6B and MCMV. Conformer a-1 and b-1 do not exist in MCMV.

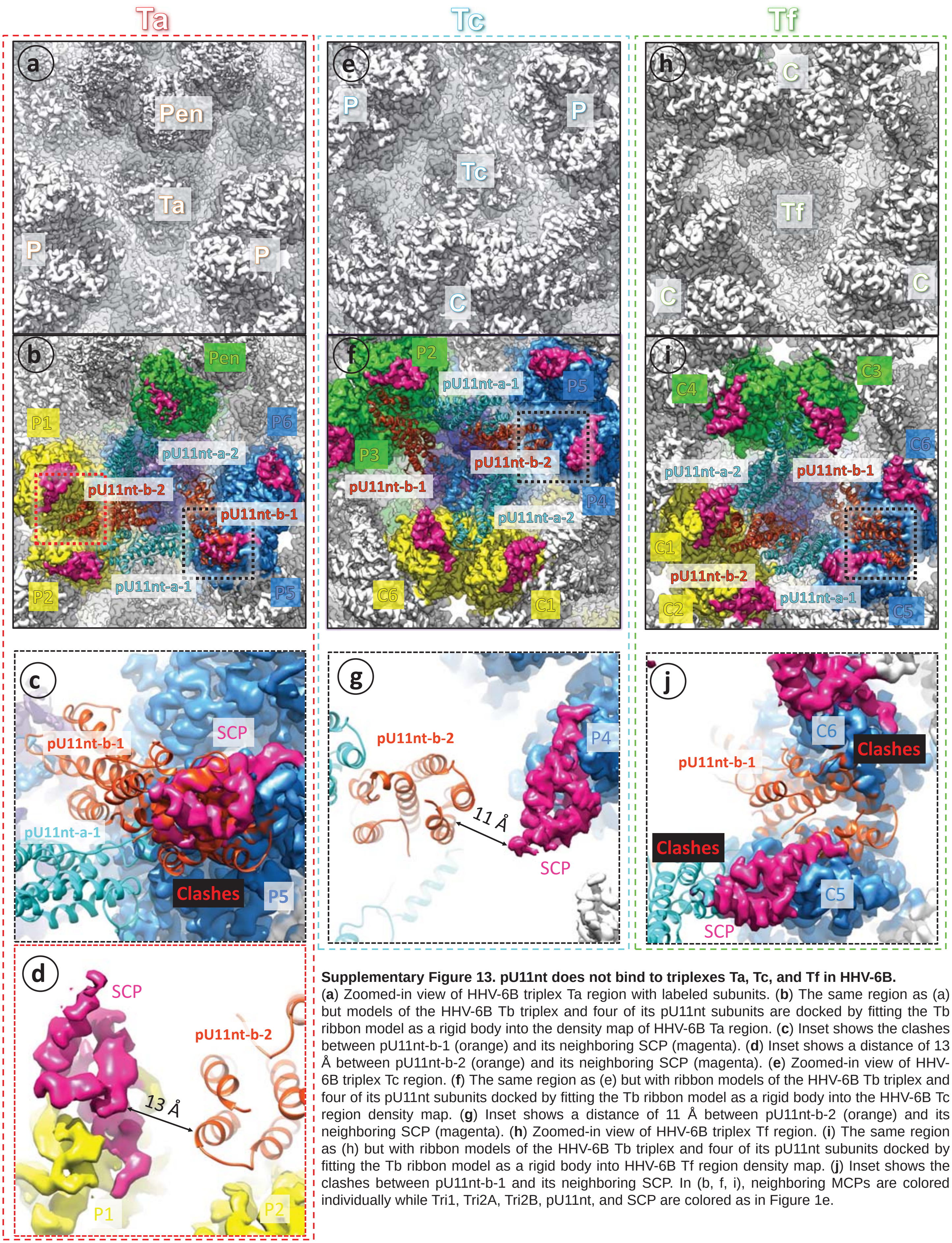

Supplementary Figure 13. pU11nt does not bind to triplexes Ta, Tc, and Tf in HHV-6B.
(**a**) Zoomed-in view of HHV-6B triplex Ta region with labeled subunits. (**b**) The same region as (a) but models of the HHV-6B Tb triplex and four of its pU11nt subunits are docked by fitting the Tb ribbon model as a rigid body into the density map of HHV-6B Ta region. (**c**) Inset shows the clashes between pU11nt-b-1 (orange) and its neighboring SCP (magenta). (**d**) Inset shows a distance of 13 Å between pU11nt-b-2 (orange) and its neighboring SCP (magenta). (**e**) Zoomed-in view of HHV-6B triplex Tc region. (**f**) The same region as (e) but with ribbon models of the HHV-6B Tb triplex and four of its pU11nt subunits docked by fitting the Tb ribbon model as a rigid body into the HHV-6B Tc region density map. (**g**) Inset shows a distance of 11 Å between pU11nt-b-2 (orange) and its neighboring SCP (magenta). (**h**) Zoomed-in view of HHV-6B triplex Tf region. (**i**) The same region as (h) but with ribbon models of the HHV-6B Tb triplex and four of its pU11nt subunits docked by fitting the Tb ribbon model as a rigid body into HHV-6B Tf region density map. (**j**) Inset shows the clashes between pU11nt-b-1 and its neighboring SCP. In (b, f, i), neighboring MCPs are colored individually while Tri1, Tri2A, Tri2B, pU11nt, and SCP are colored as in Figure 1e.

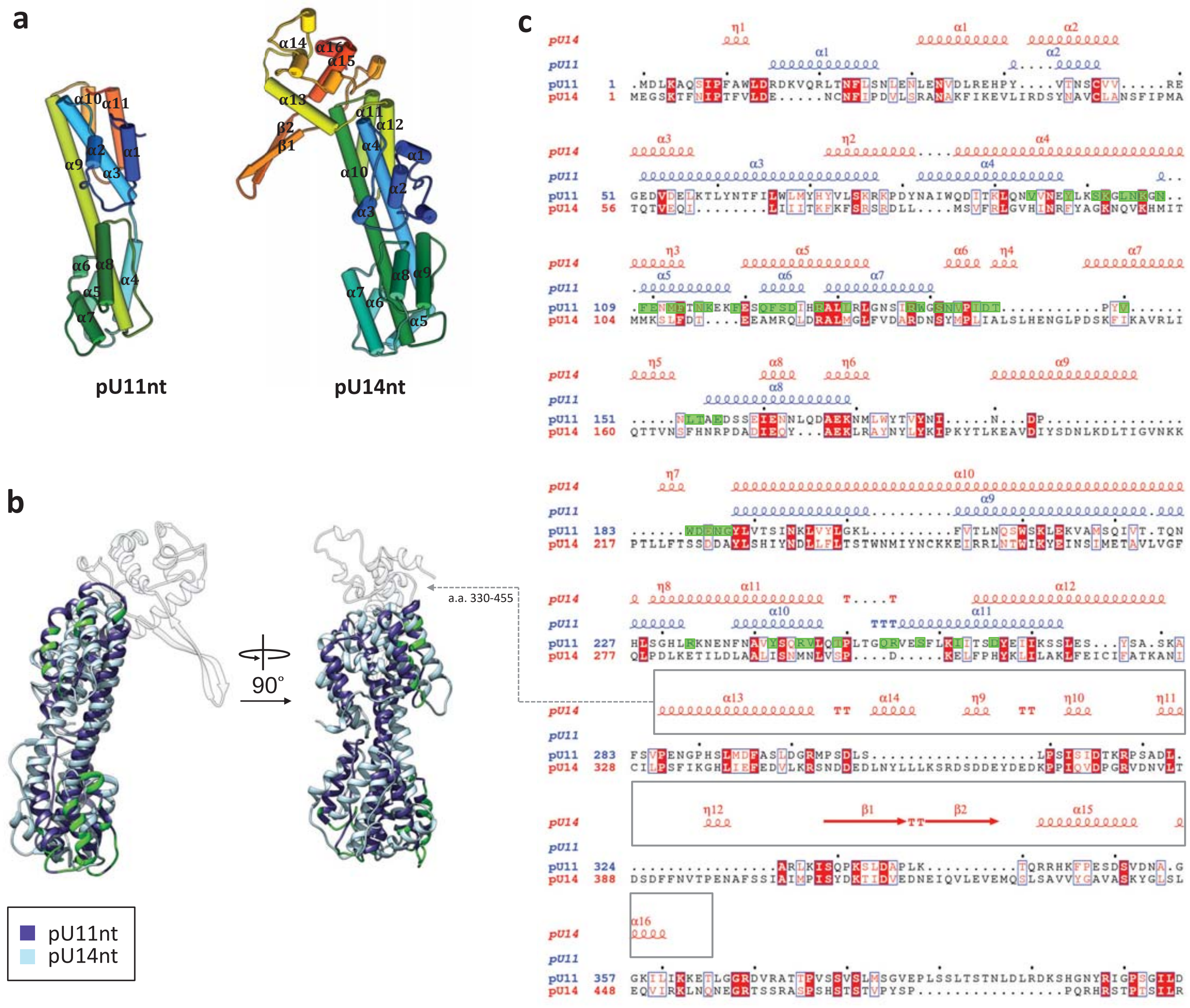

Supplementary Figure 14. pU11 shares a similar fold with pU14.

(**a**) Atomic models of N-terminal domain of pU11 (pU11nt) and pU14 (pU14nt) proteins shown as pipes and planks (blue at the N-terminus to red at the C-terminus) with their α-helixes labeled. (**b**) Superposed pU11nt (dark blue) and pU14nt (light blue) reveal that pU11nt (a.a. 1-284) and pU14nt (a.a. 1-329) share a highly similar fold. Residues 330-455 are shown as transparency for clarity. pU11nt residues within a distance of 4 Å from neighboring capsid proteins are shown in green. (**c**) Secondary structure and sequence alignment for pU11nt and pU14nt. Those pU11nt residues that are within a distance of 4 Å from neighboring capsid proteins are highlighted in green shades.

Supplementary Tables

Supplementary Table 1. Data collection summarization for HHV-6B, HSV-1, HSV-2, HCMV, and KSHV.

	HSV-1	HSV-2	HSV-2 CVSC	HCMV	HHV-6B	KSHV
Movie No.	7,356	4,600	7,902	12,000	4,828	8,007
Particle No.	45,530	55,000	50,000	39,600	6,443	44,343
Pixel size (Å)	1.03	1.38	1.41	1.61	1.085	1.03
Voltage (kV)	300	300	300	300	300	300
Detector	K2	Falcon3	Falcon3	K2	K2	K2
Data collection software	Leginon	SerialEM	SerialEM	Leginon	SerialEM	Leginon
Defocus range (μm)	1.0-3.0	0.8-2.3	0.8-2.3	2.0-2.5	2.2-3.2	1.0-3.0
Icosahedral Resolution (Å)	4.2	4.0	4.2	3.9	5.1	4.2
Sub-particle Resolution (Å)	3.5	3.1	3.7		3.8	
Reference	Ref. [18]	Ref. [19]	Ref. [20]	Ref. [16]		Ref. [17]

Supplementary Table 2. CryoEM data collection, refinement and validation statistics for HHV-6B icosahedral and sub-particle (2-fold, 3-fold, and 5-fold) reconstructions, and atomic models.

	EMD-20557	EMD-20558	EMD-20560	EMD-20559
	Capsid	2-fold	3-fold	5-fold
**Data collection and processing**				
Magnification	64,000 (super mode)			
Voltage (kV)	300			
Electron exposure (e^-/Å^2)	23			
Defocus range (μm)	2.2-3.2			
Pixel size (Å)	1.085			
Symmetry imposed	Icosahedral	C2	C3	C5
Initial particle images (No.)	7,430			
Final particle images (No.)	6,443	196,290	128,860	77,316
Map resolution (Å)	5.1	3.77	3.77	3.82
Refinement				
Initial model used (PDB code)	N/A			
Model resolution (Å)	N/A			
Map sharpening B factor (Å^2)	205	120	120	120
**Model validation** (PDBID 6Q1F)				
Model composition				
Non-hydrogen atoms	238,592			
Protein residues	29,876			
Ligands	0			
R.M.S. deviations				
Bond lengths (Å)	0.008			
Bond angles (°)	1.146			
Validation				
MolProbity score	2.07			
Clash score	12.14			
Poor rotamers (%)	0.79			
Ramachandran plot				
Favored (%)	92.37			
Allowed (%)	7.32			
Disallowed (%)	0.31			

Supplementary Table 3. Comparison of capsid, pU11/pUL32, pU14/pUL25 proteins in HHV-6B and in HCMV.

Protein names	Size (a.a.)		Sequence (%)	
	HCMV (AD169 strain)	HHV-6B (Z29 strain)	Identity	Similarity
**MCP**	1370	1345	43.9	67.0
**SCP**	75	89	30.4	45.7
**Tri1**	290	299	28.6	50.0
**Tri2**	306	296	42.6	61.5
**pUL32/pU11**	1048	858	14.9	25.1
**pUL25/pU14**	656	610	13.9	24.3

Supplementary References

1. Kucukelbir, A., Sigworth, F.J. & Tagare, H.D. Quantifying the local resolution of cryo-EM density maps. *Nature Methods* **11**, 63 (2013).
2. Wang, B. et al. Crystal Structure of Human Herpesvirus 6B Tegument Protein U14. *PLOS Pathogens* **12**, e1005594 (2016).
3. Zhang, E. et al. Inherited Chromosomally Integrated Human Herpesvirus 6 Genomes Are Ancient, Intact, and Potentially Able To Reactivate from Telomeres. *Journal of Virology* **91**, e01137-01117 (2017).
4. Megaw, A.G., Rapaport, D., Avidor, B., Frenkel, N. & Davison, A.J. The DNA Sequence of the RK Strain of Human Herpesvirus 7. *Virology* **244**, 119-132 (1998).
5. Bradley, A.J. et al. High-throughput sequence analysis of variants of human cytomegalovirus strains Towne and AD169. *Journal of General Virology* **90**, 2375-2380 (2009).
6. Rawlinson, W.D., Farrell, H.E. & Barrell, B.G. Analysis of the complete DNA sequence of murine cytomegalovirus. *J Virol* **70**, 8833-8849 (1996).
7. Vink, C., Beuken, E. & Bruggeman, C.A. Complete DNA Sequence of the Rat Cytomegalovirus Genome. *Journal of Virology* **74**, 7656 (2000).
8. Davison, A.J. et al. The human cytomegalovirus genome revisited: comparison with the chimpanzee cytomegalovirus genomeFN1. *Journal of General Virology* **84**, 17-28 (2003).
9. Baxter, M.K. & Gibson, W. Cytomegalovirus basic phosphoprotein (pUL32) binds to capsids in vitro through its amino one-third. *J Virol* **75**, 6865-6873 (2001).
10. Blewett, E.L., Sherrod, C.J., Texier, J.R., Conrad, T.M. & Dittmer, D.P. Complete Genome Sequences of Mandrillus leucophaeus and Papio ursinus Cytomegaloviruses. *Genome announcements* **3**, e00781-00715 (2015).
11. Hansen, S.G., Strelow, L.I., Franchi, D.C., Anders, D.G. & Wong, S.W. Complete Sequence and Genomic Analysis of Rhesus Cytomegalovirus. *Journal of Virology* **77**, 6620 (2003).
12. Davison, A.J. et al. Comparative genomics of primate cytomegaloviruses, Vol. 1. (2013).
13. Marsh, A.K. et al. Genomic Sequencing and Characterization of Cynomolgus Macaque Cytomegalovirus. *Journal of Virology* **85**, 12995 (2011).
14. Yang, D. et al. Complete genome sequence of pathogenic Guinea pig cytomegalovirus from salivary gland homogenates of infected animals. *Genome announcements* **1**, e0005413-e0005413 (2013).
15. Robert, X. & Gouet, P. Deciphering key features in protein structures with the new ENDscript server. *Nucleic Acids Research* **42**, W320-W324 (2014).
16. Yu, X., Jih, J., Jiang, J. & Zhou, Z.H. Atomic structure of the human cytomegalovirus capsid with its securing tegument layer of pp150. *Science* **356** (2017).
17. Dai, X. et al. Structure and mutagenesis reveal essential capsid protein interactions for KSHV replication. *Nature* **553**, 521 (2018).
18. Dai, X. & Zhou, Z.H. Structure of the herpes simplex virus 1 capsid with associated tegument protein complexes. *Science* **360** (2018).
19. Yuan, S. et al. Cryo-EM structure of a herpesvirus capsid at 3.1 Å. *Science* **360**, eaao7283 (2018).
20. Wang, J. et al. Structure of the herpes simplex virus type 2 C-capsid with capsid-vertex-specific component. *Nature Communications* **9**, 3668 (2018).
