# Supplementary material for: Atomic structure of the human herpesvirus 6B capsid and capsid-associated tegument complexes
Source: Nat Commun. 2019 Nov 25;10:5346. doi: 10.1038/s41467-019-13064-x (PMC6877594; doi:10.1038/s41467-019-13064-x)
Supplement: Supplementary file 2 — Description of Additional Supplementary Files [file 41467_2019_13064_MOESM2_ESM.pdf]

## Description of Additional Supplementary Files

**File name:** Supplementary Movie 1
**Description:** Shaded surface view of the icosahedral reconstruction of the HHV-6B virion. Only the capsid and associated tegument protein pU11 (pp150) are visible.

**File name:** Supplementary Movie 2
**Description:** High-resolution structure features in the 3.82 Å resolution sub-particle reconstruction from the region around the 2-fold axis.

**File name:** Supplementary Movie 3
**Description:** High-resolution structure features in the 3.77 Å resolution sub-particle reconstruction from the region around the 3-fold axis.

**File name:** Supplementary Movie 4
**Description:** High-resolution structure features in the 3.77 Å resolution sub-particle reconstruction from the region around the 5-fold axis.

**File name:** Supplementary Movie 5
**Description:** Ribbon structures showing the three types of capsid floor-defining MCP-MCP interactions in HHV-6B.

**File name:** Supplementary Movie 6
**Description:** Ribbon structures of the HHV-6B triplex Td illustrating their domains and interactions with the MCP floor.

**File name:** Supplementary Movie 7
**Description:** Interactions among the subunits of HHV-6B tegument protein pU11 tetramer (i.e., “dimer-of-dimers”) and between pU11 and capsid proteins MCP and SCP.
