# Supplementary material for: Atomic structure of the human herpesvirus 6B capsid and capsid-associated tegument complexes
Source: Nat Commun. 2019 Nov 25;10:5346. doi: 10.1038/s41467-019-13064-x (PMC6877594; doi:10.1038/s41467-019-13064-x)
Supplement: Supplementary file 10 — Reporting Summary [file 41467_2019_13064_MOESM10_ESM.pdf]

nature research

Corresponding author(s): Zhou, Z. H.

Last updated by author(s): Oct 9, 2019

Reporting Summary

Nature Research wishes to improve the reproducibility of the work that we publish. This form provides structure for consistency and transparency in reporting. For further information on Nature Research policies, see Authors & Referees and the Editorial Policy Checklist .

Statistics

For all statistical analyses, confirm that the following items are present in the figure legend, table legend, main text, or Methods section.

n/a	Confirmed	
☐	☒	The exact sample size (*n*) for each experimental group/condition, given as a discrete number and unit of measurement
☐	☒	A statement on whether measurements were taken from distinct samples or whether the same sample was measured repeatedly
☒	☐	The statistical test(s) used AND whether they are one- or two-sided<br>*Only common tests should be described solely by name; describe more complex techniques in the Methods section.*
☒	☐	A description of all covariates tested
☒	☐	A description of any assumptions or corrections, such as tests of normality and adjustment for multiple comparisons
☒	☐	A full description of the statistical parameters including central tendency (e.g. means) or other basic estimates (e.g. regression coefficient) AND variation (e.g. standard deviation) or associated estimates of uncertainty (e.g. confidence intervals)
☒	☐	For null hypothesis testing, the test statistic (e.g. *F*, *t*, *r*) with confidence intervals, effect sizes, degrees of freedom and *P* value noted<br>*Give P values as exact values whenever suitable.*
☒	☐	For Bayesian analysis, information on the choice of priors and Markov chain Monte Carlo settings
☒	☐	For hierarchical and complex designs, identification of the appropriate level for tests and full reporting of outcomes
☒	☐	Estimates of effect sizes (e.g. Cohen's *d*, Pearson's *r*), indicating how they were calculated

Our web collection on statistics for biologists contains articles on many of the points above.

Software and code

Policy information about availability of computer code

Data collection	Commercial human T lymphoblastic cell line SupT1 (ATCC CRL-1942), RPMI medium (RPMI 1640, Life Technologies), 10% heat-inactivated fetal bovine serum (FBS), 1% L-Glutamine (200nM final concentration) and 1% penicillin-streptomycin (100U/mL) were used in HHV-6B virus culture; Commercial 200-mesh Quantifoil R2/1 grids were used in HHV-6B CryoEM sample preparation; Commercial 300kV FEI Titan Krios cryo-electron microscope equipped with a Gatan imaging filter (GIF) on a Gatan K2 Summit direct electron detector were leveraged in HHV-6B image acquirement; An open source software SerialEM was used during our imaging procedure.
Data analysis	An open source software MotionCor2 was used to align and average each HHV-6B movies; An open source software CTFFIND4 was used to determine the defocus of each micrograph; An open source softwere Relion was used to perform 2D classification, 3D refinement, and 3D reconstruction for either bin4 HHV-6B viron/NIEP particles or bin1 2f/3f/5f sub-particles; An open source code Scipion was used to determine the location and recalculate the defocus of each 2f/3f/5f sub-particle; An open source web server SWISS-MODEL was used to build the homology models within one HHV-6B asymmetric unit; An open source software Phenix was used to build the HHV-6B atomic models; An open source softwere Chimera was used to realize model visualization; An open source web server wwPDB validation was used to describe the geometric outliers and report the quality of the atomic models; An open source software Coot was uesd to fix the atomic models manually.

For manuscripts utilizing custom algorithms or software that are central to the research but not yet described in published literature, software must be made available to editors/reviewers. We strongly encourage code deposition in a community repository (e.g. GitHub). See the Nature Research guidelines for submitting code & software for further information.

Data

Policy information about availability of data

All manuscripts must include a data availability statement. This statement should provide the following information, where applicable:

- Accession codes, unique identifiers, or web links for publicly available datasets
- A list of figures that have associated raw data
- A description of any restrictions on data availability

CryoEM maps and atomic models are deposited in the Electron Microscopy Data Bank (EMDB) and the RCSB Protein Data Bank (PDB), respectively. They include the cryoEM density maps of the HHV-6B capsid, sub-particle reconstructions at 2-fold, 3-fold, and 5-fold axes (accession code EMD-20557, EMD-20558, EMD-20560, and EMD-20559, respectively) and a single coordinate file containing 59 atomic models (PDB accession code 6Q1F).

Field-specific reporting

Please select the one below that is the best fit for your research. If you are not sure, read the appropriate sections before making your selection.

[x] Life sciences   [ ] Behavioural & social sciences   [ ] Ecological, evolutionary & environmental sciences

For a reference copy of the document with all sections, see nature.com/documents/nr-reporting-summary-flat.pdf

Life sciences study design

All studies must disclose on these points even when the disclosure is negative.

Sample size	Sample size of HHV-6B was determine by cryo electron microscopy
Data exclusions	None viron/NIEP or low SNR HHV-6B particles were excluded
Replication	The experiment is reproducible as long as the amount of HHV-6B particle is adequate
Randomization	The HHV-6B particle were sparsely distributed in our CryoEM grids. Areas with thicker ice have more particles
Blinding	The investigators were not blinded as we can clearly see HHV-6B particles due to its large particle size

Reporting for specific materials, systems and methods

We require information from authors about some types of materials, experimental systems and methods used in many studies. Here, indicate whether each material, system or method listed is relevant to your study. If you are not sure if a list item applies to your research, read the appropriate section before selecting a response.

Materials & experimental systems

n/a	Involved in the study	
[x]	[ ]	Antibodies
[ ]	[x]	Eukaryotic cell lines
[x]	[ ]	Palaeontology
[x]	[ ]	Animals and other organisms
[x]	[ ]	Human research participants
[x]	[ ]	Clinical data

Methods

n/a	Involved in the study	
[x]	[ ]	ChIP-seq
[x]	[ ]	Flow cytometry
[x]	[ ]	MRI-based neuroimaging

Eukaryotic cell lines

Policy information about cell lines

Cell line source(s)	SUPT1 cell line (ATCC CRL-1942)
Authentication	cell line was obatained from ATCC
Mycoplasma contamination	cell line was not tested for mycoplasma
Commonly misidentified lines (See ICLAC register)	None
